# An ultrasound assisted, ionic liquid-molecular iodine synergy driven efficient green synthesis of pyrrolobenzodiazepine-triazole hybrids as potential anticancer agents

**DOI:** 10.3389/fphar.2023.1168566

**Published:** 2023-05-05

**Authors:** Mohammad Saquib, Shakir Ahamad, Mohammad Faheem Khan, Mohammad Imran Khan, Mohd Kamil Hussain

**Affiliations:** ^1^ Department of Chemistry, University of Allahabad, Prayagraj, Uttar Pradesh, India; ^2^ Department of Chemistry, Aligarh Muslim University, Aligarh, Uttar Pradesh, India; ^3^ Department of Biotechnology, Era’s Lucknow Medical College, Era University, Lucknow, Uttar Pradesh, India; ^4^ Department of Biochemistry, Faculty of Science, King Abdulaziz University, Jeddah, Saudi Arabia; ^5^ Centre of Artificial Intelligence in Precision Medicine, King Abdulaziz University, Jeddah, Saudi Arabia; ^6^ Department of Chemistry, Govt. Raza P.G. College, Rampur, Uttar Pradesh, India; ^7^ M.J.P Rohilkahand University, Bareilly, Uttar Pradesh, India

**Keywords:** pyrrolobenzodiazepine-triazole hybrids, ionic liquid, iodine, green synthesis, anti-cancer agents

## Abstract

Herein, we report an efficient and eco-friendly, ultrasound assisted synthetic strategy for the construction of diversified pyrrolobenzodiazepine-triazole hybrids, which are potentially pharmaceutically important scaffolds, via a domino reaction involving intermolecular electrophilic substitution followed by intramolecular Huisgen 1,3-dipolar azide-alkyne cycloaddition. The USP of the reported protocol is the use of benign and inexpensive, recyclable molecular iodine-ionic liquid synergistic catalytic system cum reaction media for achieving the synthesis. The other salient features of this method are the use of mild reaction conditions, high yield and atom economy, operational simplicity, broad substrate scope and easy workup and purification. All the synthesized compounds were evaluated for *in vitro* anti-proliferative activity against various cancer cell lines. From among the synthesized title compounds, 9,9-dimethyl-8-phenyl-9H-benzo [b]pyrrolo [1,2-d][1,2,3]triazolo[5,1-g][1,4]diazepine (7) was found most to be the most active compound exhibiting IC_50_ value of 6.60, 5.45, 7.85, 11.21, 12.24, 10.12, and 11.32 µM against MCF-7, MDA-MB-231, HeLa, SKOV-3, A549, HCT-116 and DLD-1 cell lines, respectively. Further the compounds were found to be non-toxic against normal human embryonic kidney (HEK-293) cell line.

## Introduction

The classical methods for the construction of diverse fused heterocyclic compounds entail the sequential formation of the individual bonds in the target molecule. However, a more effective strategy is to form the various bonds in a single operation via domino/cascade or multi-component reactions (MCR), without separating the intermediates, altering the reaction conditions, leading to faster synthesis and low waste production, thus drastically reducing the amount of solvent, reagents, energy and labor used, as well as simplifying the purification process (Tietze, 1996; Lu et al., 2012; Cioc et al., 2014). Due to the mounting awareness for a sustainable future in the last 2 decades the use of eco-friendly techniques and processes, reagents, solvents and catalysts in organic syntheses has become imperative (Li and Trost, 2008; Sheldon, 2008; Tiwari et al., 2018; Rather and Ali, 2021, 2022; Kar et al., 2022; Saquib et al., 2022). In this milieu domino/cascade strategy assumes even greater relevance and has become the one of the strategies of choice for the synthesis of medicinally significant molecules (Masson, 2014; Ahamad et al., 2016a, 2016b, 2018; Xie et al., 2021; Hussain et al., 2022). Conventional volatile organic compound (VOC) based solvents and metal based toxic catalysts are responsible for the major part of waste emanating from chemical processes and industries. Thus, the designing of an effective eco-friendly synthesis cannot be possible without the replacement of conventional VOC based solvents and toxic catalysts with eco-friendly solvents and catalysts (Marafi and Stanislaus, 2008; Menges, 2018).

Molecular iodine has received considerable attention as an inexpensive, non-toxic, readily available catalyst for various organic transformations, affording the corresponding products in excellent yields with high selectivity. The mild Lewis acidity associated with iodine enhanced its usage in organic synthesis to realize several organic transformations using stoichiometric levels to catalytic amounts of iodine. Due to these reasons iodine has been used as a green catalyst for dif-ferent organic reactions (Yusubov and Zhdankin, 2015; Breugst et al., 2016; Marsili et al., 2018; Tran et al., 2019; Velasco et al., 2022; Monika et al., 2023). Likewise, ionic liquids (ILs) are a new and versatile class of compounds with immense utility across many spheres of scientific research. Their unique eco-friendly physical and chemical properties are increasingly attracting chemists to explore their use as a green reaction media for organic synthesis. ILs offer a variety of advantages over conventional organic solvents, for instance product isolation is easier, they can be reused and catalysts can be more easily recovered and recycled, they are usually not flammable, vapor pressure is very low, and very importantly they have good ability to dissolve organic, organometallic, and even many inorganic compounds. Consequently, over the past decade or so, there has been a dramatic increase in the number of publications in the field of ionic liquids. [Bmim]BF_4_ is an inexpensive and commercially available, efficient, less toxic and recyclable ILs that has been successfully used in a variety of organic transformations. It has been reported to be superior to other commonly used imidazolium based ILs (Earle and Seddon, 2000; Rogers and Seddon, 2003; Ren and Cai, 2010; Banerjee, 2017; Dong et al., 2017; Gupta P. K. et al., 2022).

Cancer is one of the leading causes of mortality worldwide (Siegel et al., 2023). An estimated 19.3 million new cancer cases and more than 10 million deaths due to cancer in were reported 2020. Breast cancer is now the most commonly diagnosed malignancy, with a projected 2.3 million new cases (11.7%), followed by lung (11.4%), colorectal (10.0%), prostate (7.3%), and stomach (5.6%) cancers (Sung et al., 2021). The worrying rise in occurrence of new types of cancer represents a big crisis for public health systems around the world. Although a variety of anti-cancer drugs are currently available (Olivier et al., 2021) their efficacy is limited by toxicity to normal cells and drug resistance (Nurgali et al., 2018; Vasan et al., 2019). Therefore, the development of newer anti-cancer therapeutics having greater effectiveness and lesser toxicity, and with novel mechanisms of action, are urgently required (Hussain et al., 2014b; Saquib et al., 2019; Zhong et al., 2021; Gupta A. et al., 2022). Pyrrole, benzodiazepines and 1,2,3-triazoles are among the most medicinally relevant heterocyclic scaffolds (Ahamad et al., 2018; Mateev et al., 2022; Tolu-Bolaji et al., 2022).

**FIGURE 1:**
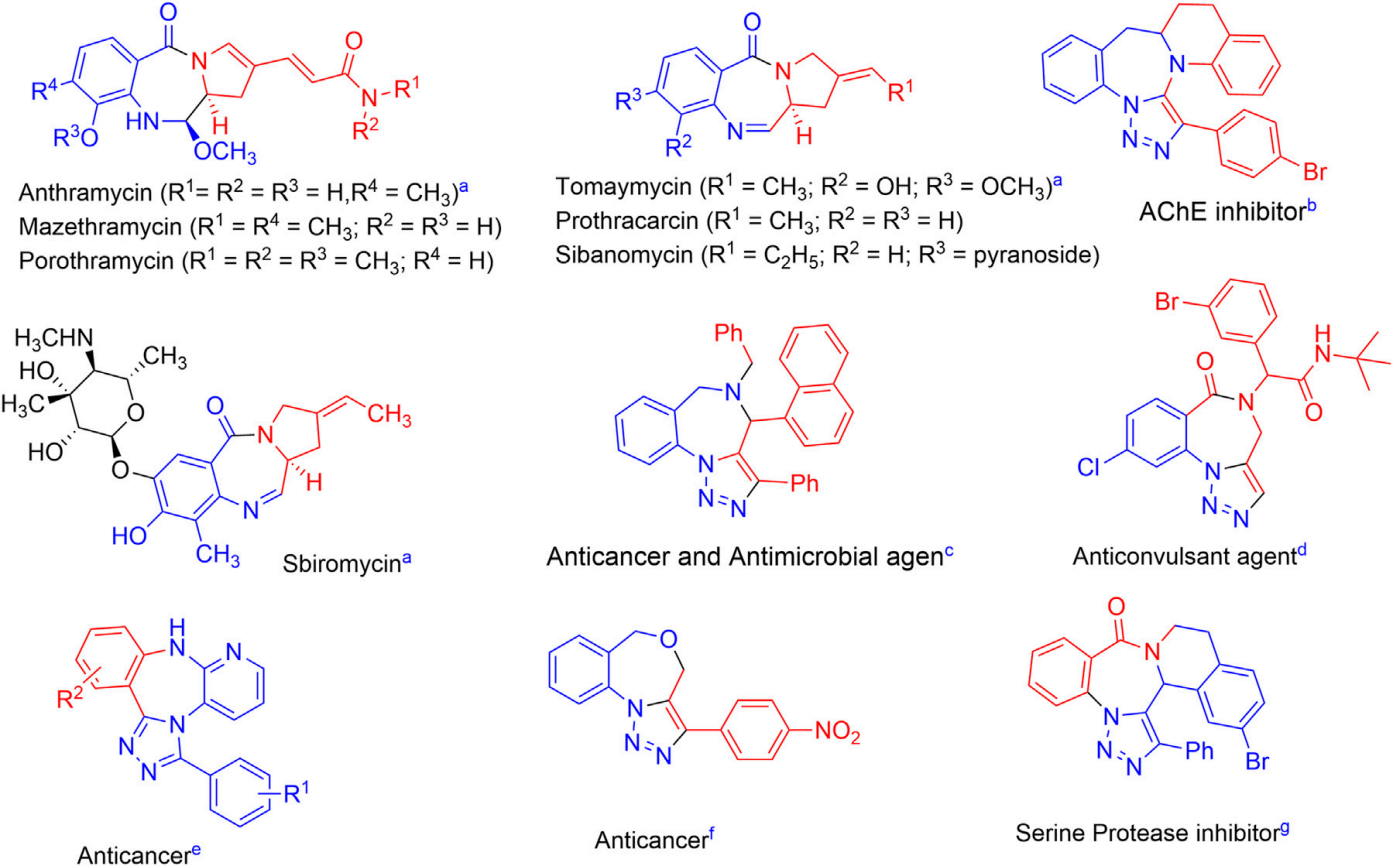
Selected medicinally active pyrrolobenzodiazepine-triazoles hybrids and related mole-cules. ^a^(Fotso, S. et al., 2009; Antonow and Thurston, 2011); ^b^(Sudhapriya et al., 2019); ^c^(Shafie et al., 2020); ^d^(Ali et al., 1990); ^e^(Banerji et al., 2013); ^f^(Granger et al., 2011); ^g^(Kamal et al., 2011).

A number of researchers have recently reported the synthesis of scaffolds in which these moieties have been fused together, many of which show good bioactivities including anti-cancer activity (Figure 1) (Ali et al., 1990; Fotso et al., 2009; Kamal et al., 2010, 2011; Antonow and Thurston, 2011; Granger et al., 2011; Banerji et al., 2013; Sudhapriya et al., 2019, 2015; Shafie et al., 2020). This result was unsurprising as it is assumed that if different active cores are integrated onto one platform, using the concept of molecular hybridization, the novel hybrid scaffold that would be obtained is expected to show enhanced medicinal properties (Meunier, 2008; Bosquesi et al., 2011; Tukulula et al., 2013; Shaveta et al., 2016).

**FIGURE 2:**
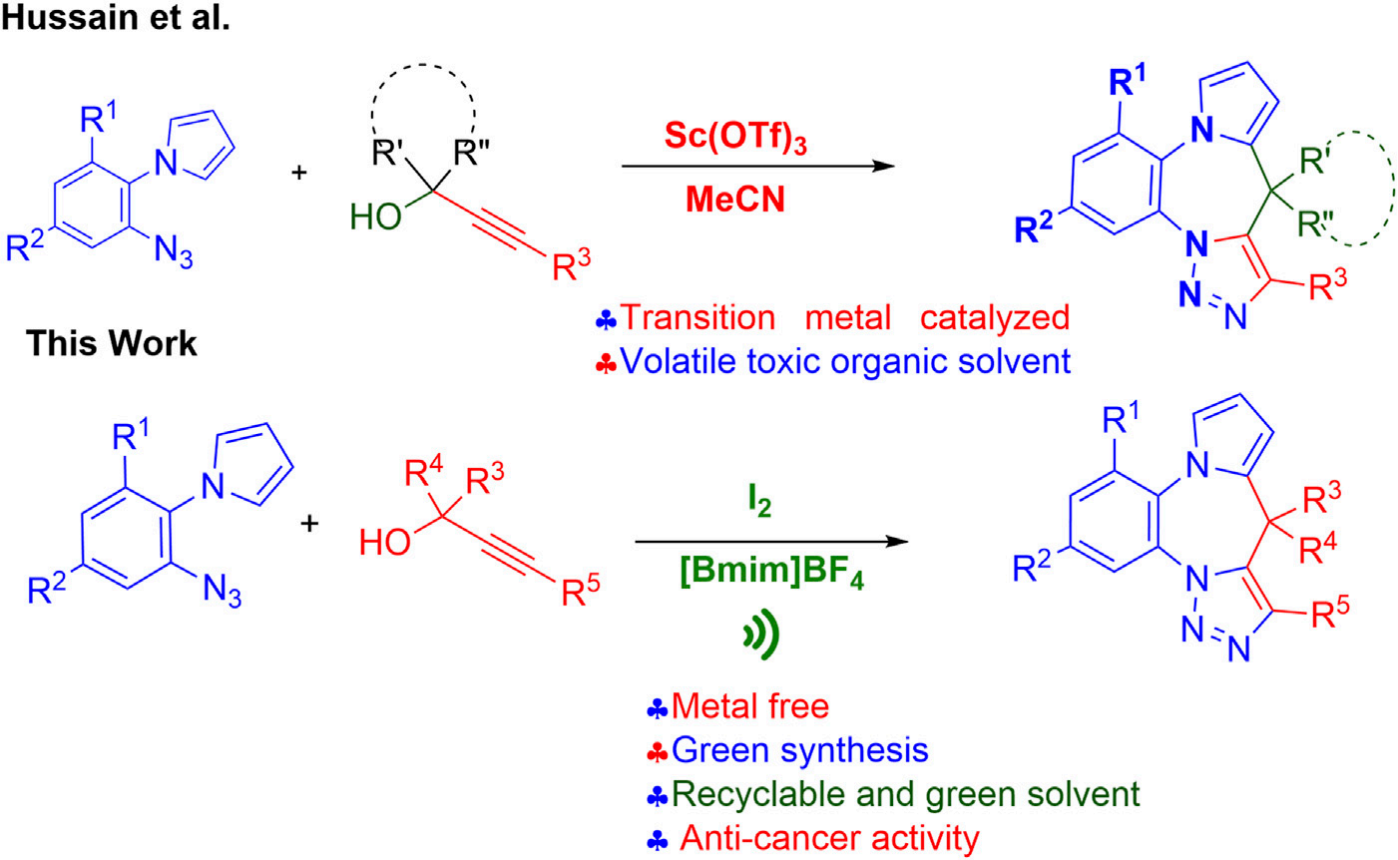
Previous and present methods for the synthesis of pyrrolobenzodiazepine-triazoles hybrids.

Working on this hypothesis we recently reported the development of a new method for the synthesis of the title compounds using scandium triflate in acetonitrile (MeCN) (Figure 2) (Hussain et al., 2014a). However, with the current emphasis on green synthesis the real challenge is the development of an eco-friendly, efficient approach for the construction of the title pyrrolobenzodiazepine-triazoles fused hybrids. In continuation of our earlier work on the design and synthesis of new hybrid scaffolds as potential bioactive agents (Ansari et al., 2015; Tiwari et al., 2017; Saquib et al., 2021) we herein report an efficient and atom-economic eco-friendly domino approach to highly diversified pyrrolobenzodiazepine-triazoles fused hybrids using a synergistic iodine-[Bmim]BF4 catalytic system, using easily accessible substrates, in the presence of ultrasonic radiation, and the *in vitro* evaluation of their anti-cancer activity against various cancer cell lines.

## Results and discussion

**FIGURE 3:**
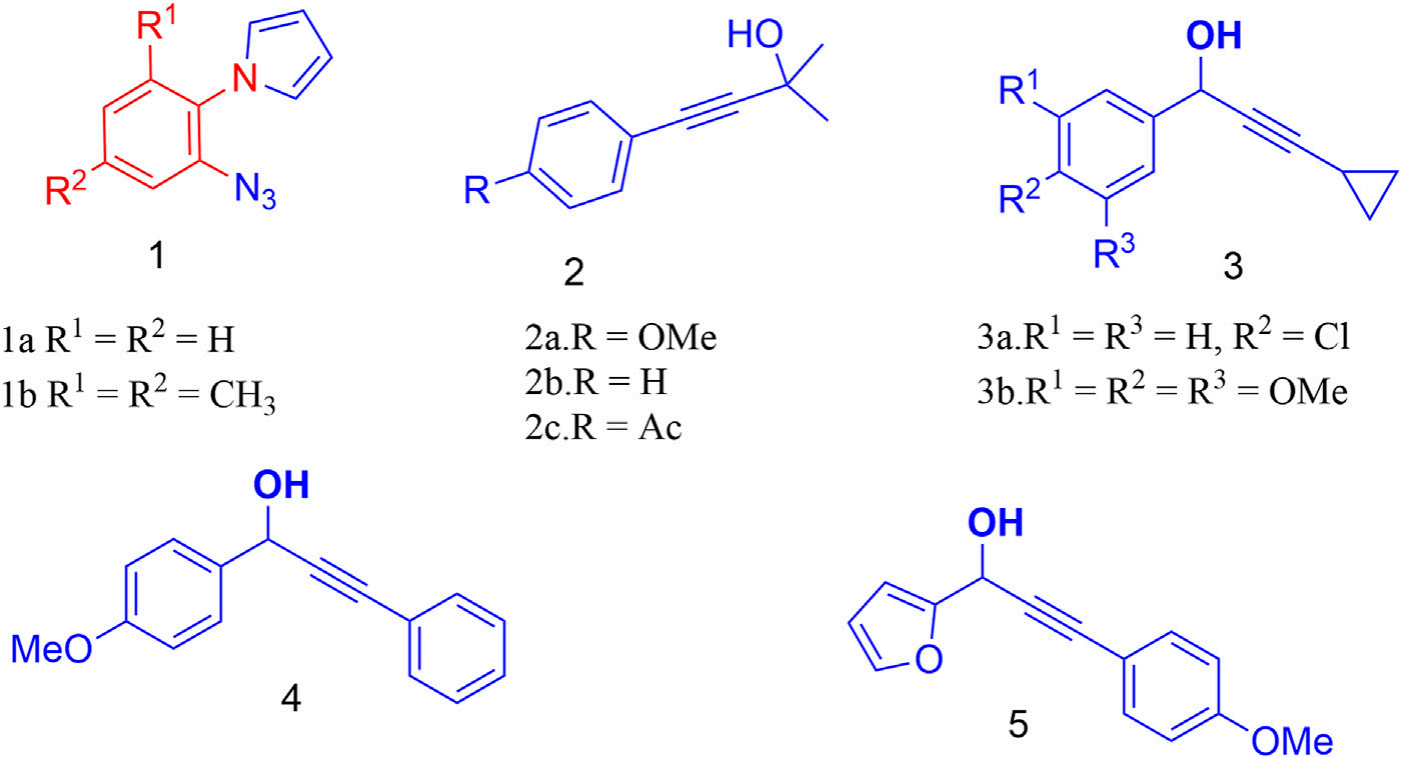
Structures of various substrates required for the construction of the target molecules.

The required precursors (Figure 3), 1-(2-azidoaryl)-1*H*-pyrroles (1a and 1b) were obtained from 2-azidoanilines through Paal-Knorr pyrrole synthesis while propargyl alcohols (2–5) were synthesized from aldehydes and terminal alkynes (Hussain et al., 2014a). With the precursors in hand, we investigated the intermolecular propargylation followed by intramolecular 1,3-dipolar cycloaddition reaction by using a variety of Bronsted and Lewis acid catalysts (Table 1). We attempted the reaction of 1-(2-azidophenyl)-1*H*-pyrrole 1a and 4-(4-methoxyphenyl)-2-methylbut-3-yn-2-ol 2a under the influence of PTSA, oxalic acid, acetic acid and triflic acid (TfOH) at temperatures ranging from RT to 80°C. However, no reaction was observed with any of these catalysts (Table 1, entries 1–4).

**TABLE 1:** Screening of various catalysts and solvents under normal stirring and under ultrasonic irradiation.

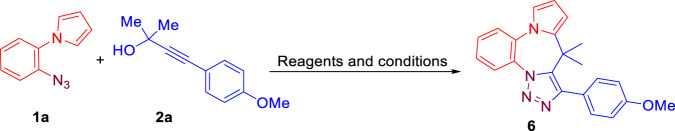					
S.N.	Catalyst/mol%	Solvent	Temperature/Time	Method	%Yield^a^
1.	PTSA/20	[Bmim]BF_4_	80°C/5 h	Stir^b^	ND^c^
2.	Oxalic acid/20	[Bmim]BF_4_	80°C/5 h	Stir	ND
3.	CH_3_COOH/20	[Bmim]BF_4_	80°C/5 h	Stir	ND
4.	TfOH/20	[Bmim]BF_4_	80°C/5 h	Stir	ND
5.	……	[Bmim]BF_4_	RT/5 h	Stir	ND
6.	…….	[Bmim]BF_4_	80°C/5 h	Stir	37
7.	I_2_/10	[Bmim]BF_4_	RT→80°C/3 h	Stir	70
8.	I_2_/15	[Bmim]BF_4_	RT→80°C/3 h	Stir	70
9.	I_2_/5	[Bmim]BF_4_	RT→80°C/3 h	Stir	56
10.	I_2_/10	[Bmim]BF_4_	RT→40°C/4 h	Stir	70
11.	**I**_**2**_ **/10**	**[Bmim]BF**_**4**_	**RT→40**°C**/2h**	**US** ^d^	**90**
12.	I_2_/10	[Bmim]BF_4_	RT→80°C/2 h	US	90
13.	I_2_/10	[Bmim]PF_6_	RT→40°C/2 h	US	75
14.	I_2_/10	[Bmim]OH	RT→40°C/3 h	US	50
15.	I_2_/10	[Bmim]Cl	RT→40°C/3 h	US	62
16.	I_2_/10	[Bmim]Br	RT→40°C/3 h	US	70
17.	I_2_/10	[Bmim]ClO_4_	RT→40°C/3 h	US	60
18.	I_2_/10	[Bmim]OAc	RT→40°C/3 h	US	58
19.	I_2_/50	MeNO_2_	RT→40°C/3 h	US	40
20.	I_2_/50	MeCN	RT→40°C/2 h	US	52
21.	InCl_3_/10	[Bmim]BF_4_	RT→40°C/2 h	US	68
22.	AgSbF_6_/10	[Bmim]BF_4_	RT→40°C/2 h	US	62

^a^ Isolated yield.

^b^ Stirring.

^c^ Not detected.

^d^ Ultrasound.

When this reaction was carried at room temperature (RT) in [bmim][BF_4_], no reaction was observed (TLC) (Table 1, entry 6). However, when the experiment was repeated at 80°C, a new spot was observed on TLC after 5 h of stirring. The compound was isolated in about 37% yield and identified as the desired pyrrolobenzodiazepine-triazoles hybrid, 8-(4-methoxyphenyl)-9,9-dimethyl-9H-benzo [b]pyrrolo [1,2-d][1,2,3]triazolo[5,1-g][1,4]diazepine (6). Encouraged by this positive result we increased the reaction temperature (90°C) and the reaction time but a better result was not observed. We now decided to use molecular iodine as a catalyst in the reaction, Initially, 10 mol% iodine was used. Addition of iodine was done at RT and the reaction temperature was then increased to 80°C. After 3 h, the desired product was obtained in 70% yield (Table 1, entry 7). Stirring for a longer time did not result in any improvement in the yield. To optimize the yield of reaction we increased the catalyst loading to 15 mol%; however, no increase in the yield was observed (Table 1, entry 8). On the other hand, decreasing the catalyst loading to 5 mol% caused a substantial decrease in yield (56%) (Table 1, entry 9). In our effort to further increase the yield of the desired compound **6** we decided to carry out the reaction in presence of ultrasonic irradiation. Consequently, when the reaction was carried out in the presence of 10 mol% iodine, followed by use of ultrasonic irradiation a large enhancement in yield (90%) and faster reaction time was observed (Table 1, entry 11).

**FIGURE 4:**
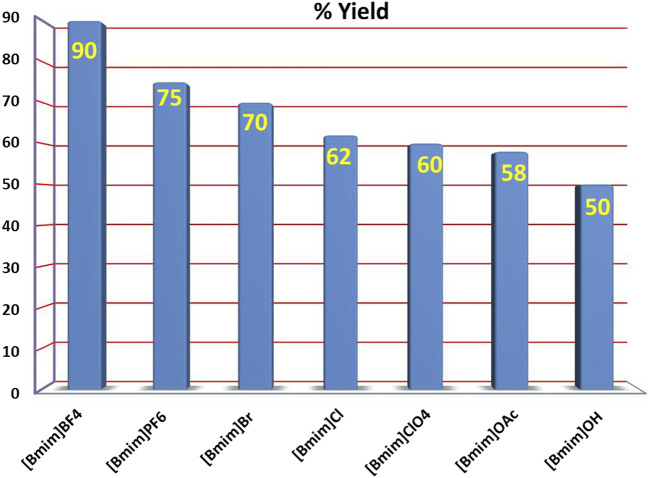
Comparison of efficacy of various ionic liquids as reaction medium for this reaction.

We also investigated the effect of different ionic liquids such as [Bmim]PF_6_, [Bmim]OH, [Bmim]Cl, [Bmim]Br, [Bmim]ClO_4_ and [Bmim]OAc on the yield and rate of the reaction. However, none of the other ionic liquids screened were found to be as effective as [Bmim]BF_4_. The effectiveness of these ionic liquids for the above reaction was observed to be in the order [bmim][BF_4_] > [Bmim]PF_6_ > [Bmim]Br >[Bmim]Cl > [Bmim]ClO_4_ > [Bmim]OAc > [Bmim]OH (Table 1, entries 13–18; Figure 4). The yield of the reaction was also found to be very low in VOC based solvents such as MeNO_2_ and MeCN (Table 1, entries 19, 20). Other Lewis acid catalysts such as InCl_3_ and AgSbF_6_ were also used but were found to be less efficient as compared to iodine under the optimized reaction conditions (Table 1, entries 21, 22). From the above set of experiments the use of 10 mol% iodine, using [Bmim]BF_4_ as the reaction medium at 40°C followed by ultrasonic irradiation of the reaction mixture was identified as the best condition for carrying out this reaction (Table 1, entry 11).

**FIGURE 5:**
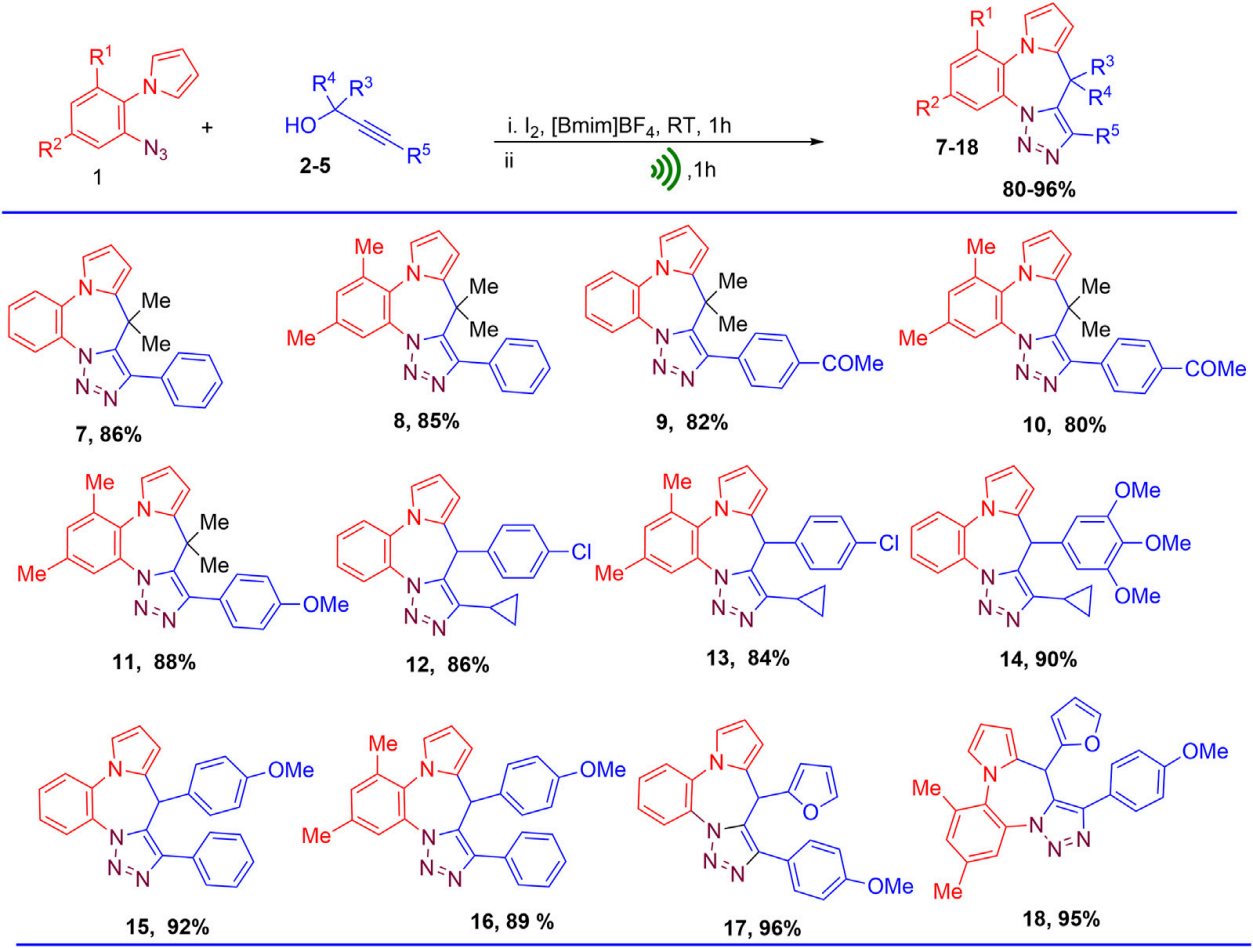
Substrate scope for the synthesis of pyrrolobenzodiazepine-triazoles hybrids (9H-benzo[b]pyrrolo[1,2-d][1,2,3]triazolo[5,1-g][1,4]diazepines).

To investigate the scope of this protocol, two 1-(2-azidoaryl)-1H-pyrroles (1a and 1b) anilines and a variety of substituted propargyl alcohols (2–5) were used as substrates to obtain the corresponding pyrrolobenzodiazepine-fused triazole hybrids. The corresponding 9*H*-benzo [b]pyrrolo [1,2-d][1,2,3]triazolo[5,1-g][1,4]diazepines were obtained in good to excellent yields. The disclosed protocol was also found to work well with both aliphatic propargyl alcohols and aromatic ring bearing propargyl alcohols having electron donating and electron withdrawing functionalities (Scheme 3). Use of propargyl alcohols with electron releasing groups at para position of the phenyl ring attached to alkynyl carbon gave better yields (Compounds 11, 17, and 18) as compared to propargyl alcohols having electron withdrawing groups (9–10). Presence of electron releasing group at para position of phenyl ring attached to C-1 carbon of propargyl alcohols also greatly assisted in the product formation leading to enhanced yield of the products (Compounds 14–16). Furan ring at 1-position of propargyl alcohols also enhanced the yield of the product (Compounds 21–22). Presence of methyl substituents at 2 and 3 position of 1-(2-azidoaryl)-1H-pyrroles led to slightly lower yields (Compounds 8, 10, 11, and 13) (Figure 5).

### Proposed mechanism

**FIGURE 6:**
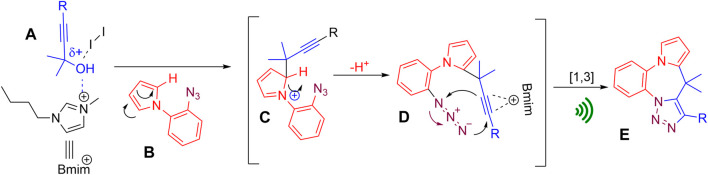
Plausible mechanism.

The reaction is assumed to initiate through the activation of alcohol by iodine and Bmim+ by forming an interaction with the oxygen atom of the propargyl alcohol A. In the presence of strong π-nucleophile pyrrole B, an electrophilic substitution reaction then takes place at the C-2 position of the pyrrole ring (the most nucleophilic center on the pyrrole nucleus) to form an intermediate species C, which undergoes re-aromatization to form C-2 propargylated pyrrole D. Ultrasonic irradiation is thought to assist the intramolecular azide−alkyne 1,3-dipolar cycloaddition reaction in intermediate D furnishing the target molecule E (Figure 6).

### Recyclability studies

**FIGURE 7:**
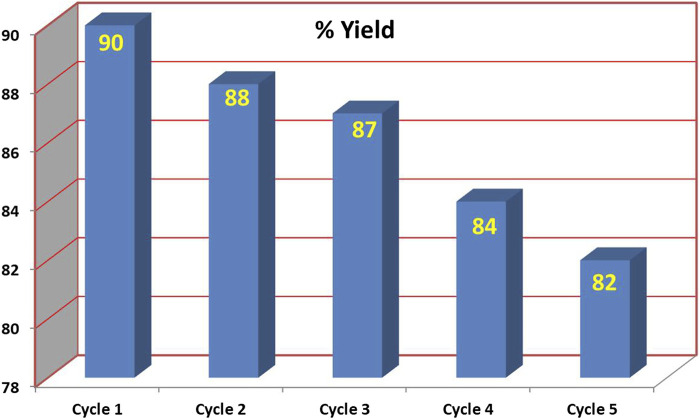
Recyclability study on [Bmim]BF_4_.

We also investigated the recyclability potential of [Bmim]BF_4_ for the formation of 8-(4-methoxyphenyl)-9,9-dimethyl-9*H*-benzo [b]pyrrolo [1,2-d][1,2,3]triazolo[5,1-g][1,4]diazepine 6 using the reaction of 1-(2-azidophenyl)-1H-pyrrole 1a with 4-(4-methoxyphenyl)-2-methylbut-3-yn-2-ol 2a. After completion of the reaction, the reaction mixture was extracted thrice with ethyl acetate. The ionic liquid residue was washed with hexane and dried in vacuum to obtain pure [bmim][BF_4_] which was used for the next cycle. The recycled Bmim][BF_4_] so obtained could be used for the reaction up to four cycles with only a small drop in yield (Figure 7).

Taking into consideration the potential medicinal significance of the title molecules we ex-amined the scale-up potential of the present protocol. Two representative reactions were performed on a gram-scale (Table 2). In both the instances the scale-up reaction was fully successful leading to the desired target molecules 6 and 7 in 90% and 86% yields, respectively (Table 2). The successful scale-up of the reaction clearly demonstrates the suitableness of this protocol on a gram-scale for further anti-cancer studies.

**TABLE 2:** Scale-up potential^a^.

Entry	Reactants		Reaction Conditions	Time (h)	Product	Yield (%)^b^
1	1a	2a	[Bmim]BF_4,_ RT→40°C/US	2	6	90
2	1a	2b	[Bmim]BF_4,_ RT→40°C/US	2	7	86

^a^ Reaction conditions: 1-(2-azidoaryl)-1H-pyrroles 1a (20 mmol) and propargyl alcohol (25 mmol) were stirred in 10 ml [Bmim]BF_4_ in a round bottom flask and then subjected to ultrasonic irradiation.

^b^ Isolated yield of product. US, Ultrasonic irradiation.

### *In vitro* antiproliferative activities of the synthesized compounds

**FIGURE 8:**
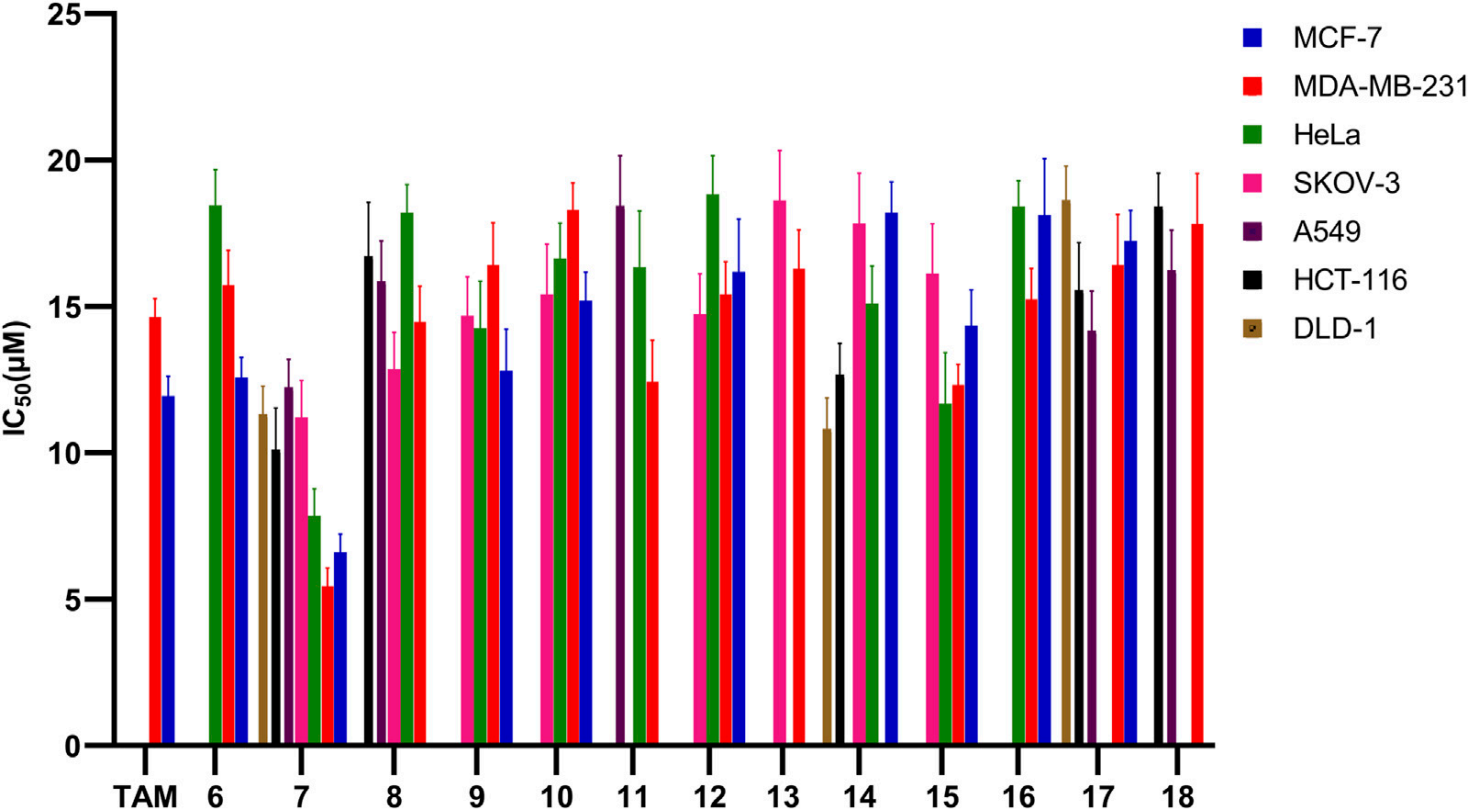
Graphical representation of calculated IC_50_ values (<19 µM) for compounds 6 to 18 against a panel of eight cancer cell-lines.

We evaluated all the molecules of the synthesized library against various cancer cell lines such as MCF-7 (ER +ve breast cancer), MDA-MB-231 (ER -ve breast cancer), HeLa (cervical cancer), SKOV-3 (ovarian cancer), A549 (lung cancer), HCT-116 and DLD-1 (colon cancer) in order to test their anti-proliferative potential (Table 3; Figure 8). Toxicity of the molecules was also evaluated against normal human embryonic kidney (HEK-293) cell line through MTT assay [35–39]. Among the evaluated molecules, compound 7 exhibited excellent activity against various cancer cells and was found to be non-toxic against HEK-293 cells. Compound 7 showed maximum anti-proliferative activity against MDA-MB-231 (IC_50_ = 5.45 µM). Compound 7 also exerted significant anti-proliferative effect against MCF-7 and Hela cell line with IC_50_ values of 6.60 and 7.85 µM respectively. Compound 7 also exhibited good anti-cancer activity against SKOV-3, A549, HCT-116 and DLD-1 cell lines with IC_50_ of 11.21, 12.24, 10.12 and 11.32 µM respectively. Compound 6 exhibited moderate anti-proliferative against MCF-7, MDA-MB-231 and HeLa cell lines with IC_50_ of 12.58, 15.72, and 18.45 µM, respectively, and was found to be inactive against SKOV-3, A549, HCT-116 and DLD-1 cell lines. Compound 8 showed activity against SKOV-3 cell line (IC_50_ = 12.86 µM). Compound 8 also exhibited mild activity against MCF-7 (IC_50_ = 19.28 µM), MDA-MB-231 (IC_50_ = 14.48 µM), HeLa (IC_50_ = 18.30 µM), A549 (IC_50_ = 15.86 µM) and HCT-116 (IC_50_ = 16.72 µM) cell lines. Compound 12 was found inactive against DLD-1 cell line. Compounds 9, 10, 12, 15, and 16 showed good to mild activity against MCF-7, MDA-MB-231, HeLa and SKOV-3 cell lines in an IC_50_ range of 12.80–19.10 µM. These compounds were found inactive against A549, HCT-116 and DLD-1 cell lines. Compounds 13 and 14 exhibited mild activity against MCF-7 and SKOV-3 cell lines. Compound 14 exhibited good activity against HCT-116 and DLD-1 cell lines with the IC_50_ value of 12.68 and 10.82 µM. Compound 14 also showed moderate inhibition against HeLa cell line (IC_50_ = 15.10 µM) and was found inactive against MDA-MB-231 and A549 cell lines. Compound 11 exhibited good activity against MDA-MB-231(IC_50_ = 12.48 µM), mild activity against HeLa (IC_50_ = 12.48 µM) and weak activity against A549 (IC_50_ = 18.44 µM) cell lines. Compounds 17 and 18 were found inactive against HeLa and SKOV-3 cell lines while in other cell lines these compounds exhibited mild to weak inhibition. From the above discussion it is clear that compound 7 having un-substituted phenyl ring attached to the triazole moiety exhibited the highest anti-cancer activity. Introduction of methyl substituents on the fused benzene ring greatly reduced the anti-cancer activity (compound 7 and 8, Table 3, entries 3 and 4). Further introduction of electron withdrawing group (compound 9 and 10) and electron releasing groups (compounds 11, 17, and 18) also decrease the activity against cancer cell lines. Introduction of cyclopropyl ring at the triazole moiety did not yield molecules with good bioactivity (compounds 12–14). Introduction of aryl ring on diazepine moiety by replacing the gem-dimethyl substituents was also found ineffective as these compound exhibited mild activity against the various cancer cell lines (compounds 12–18).

**TABLE 3:** *In vitro* antiproliferative activity of pyrrolobenzodiazepine-triazoles (6–18), against various cancer cell lines.

		IC_50_ (Mean ± SEM, in μM)							
S.No.	Comp.	MCF-7	MDA-MB-231	HeLa	SKOV-3	A549	HCT-116	DLD-1	HEK-293
1.	6	12.58 **±** 1.36	15.72 **±** 2.40	18.45 ± 2.45	>20	>20	>20	>20	>20
2.	**7**	**6.60 ± 1.25**	**5.45 ± 1.24**	**7.85 ± 1.86**	**11.21 ± 2.52**	**12.24 ± 1.92**	**10.12 ± 2.84**	**11.32 ± 1.92**	**>20**
3.	8	19.28 **±** 3.58	14.48 **±** 2.42	18.20 **±** 1.92	12.86 **±** 2.52	15.86 **±** 2.76	16.72 **±** 3.68	>20	>20
4.	9	12.80 **±** 2.86	16.42 **±** 2.87	14.26 **±** 3.22	14.68 **±** 2.68	>20	>20	>20	>20
5.	10	15.20 **±** 1.95	18.30 **±** 1.84	16.64 **±** 2.42	15.42 **±** 3.42	>20	>20	>20	>20
6.	11	>20	12.42 **±** 2.86	16.34 **±** 3.85	>20	18.44 **±** 3.42	>20	>20	>20
7.	12	16.18 **±** 3.62	15.42 **±** 2.22	18.83 **±** 2.64	14.74 **±** 2.76	>20	>20	>20	>20
8.	13	19.10 **±** 1.92	16.28 **±** 2.68	>20	18.62 **±** 3.42	>20	>20	>20	>20
9.	14	18.20 **±** 2.12	>20	15.10 **±** 2.58	17.84 **±** 3.42	>20	12.68 **±** 2.12	10.82 **±** 2.12	>20
10.	15	14.34 **±** 2.46	12.32 **±** 1.42	11.68 **±** 3.48	16.12 **±** 3.42	>20	>20	>20	19.12 **±** 1.24
11.	16	18.12 **±** 3.87	15.24 **±** 2.12	18.42 **±** 1.76	19.11 **±** 2.65	>20	>20	>20	>20
12.	17	17.23 **±** 2.12	16.42 **±** 3.45	>20	>20	14.18 **±** 2.69	15.56 **±** 3.25	18.63 **±** 2.34	>20
13.	18	19.45 **±** 1.62	17.82 **±** 3.45	>20	>20	16.24 **±** 2.74	18.42 **±** 2.26	19.10 **±** 3.26	>20
14.	TAM^a^	11.94 **±** 1.36	14.64 **±** 1.26	ND	ND	ND	ND	ND	ND

^a^ Tamoxifen.

## Experimental

### General procedure for synthesis of pyrrolobenzodiazepine-fused triazoles

To a mixture of 1-(2-azidoaryl)-1H-pyrroles 1 (2 mmol) and propargyl alcohol 2–5 (2.5 mmol) and in [bmim][BF4] (4 ml) in a round bottom flask was added a catalytic amount of iodine (10 mol%) at room temperature, and the reaction temperature was allowed to rise to 40°C. The reaction was stirred at this temperature for the next 1 h and then it was subjected to ultrasound irradiation till completion of the reaction. The reaction mixture was now quenched with addition of water and extracted thrice with 10 ml of ethyl acetate. The combined organic extracts were treated with an aqueous solution of Na_2_S_2_O_3_ (1 M), washed with saturated solution of NaHCO_3_ and dried *in vacuo* to afford the crude product mixture, which was purified through column chromatography (EtOAc/hexane). The ionic liquid residue was washed with hexane and dried in vacuum to recover the [bmim][BF_4_] which was used in the next cycle.

### Cell proliferation assay (MTT assay) in cancer cell lines and normal cell line

The anti-cancer activity of the synthesized pyrrolobenzodiazepine-triazoles hybrids against various cancer cell lines was evaluated using MTT (3-(4,5-dimethylthiazol-2-yl)-2,5-diphenyl tetrazolium bromide) reduction assay (Ansari et al., 2015; Saquib et al., 2021). 2.5 × 103 cells per well were seeded in a 100 µl Dulbecco’s modified eagle’s medium (DMEM) mixed with 10% fetal bovine serum (FBS), in each well of 96-well microculture plates and incubated at 37°C for 24 h in carbon dioxide (CO_2_) incubator. The compounds were then diluted to the desired concentrations in the culture medium. After 48 h, the media was removed and 100 µl MTT (0.5 mg/ml) solution was added to each well, and the plates were further incubated for another 3 h. Supernatant from each well was removed, formazan crystals were dissolved in 100 µl of dimethyl sulfoxide (DMSO) and absorbance was recorded at 540 nm wavelength using a spectrophotometer.

## Conclusion

In conclusion, a green and efficient domino synthetic strategy for the C-2 selective propargylation of pyrroles followed by intramolecular azide-alkyne Huisgen 1,3-dipolar cycloaddition leading to the construction of diversified pyrrolobenzodiazepine-fused triazole hybrids in high yields is reported. To the best of our information this is the first eco-friendly method for the synthesis of these biologically relevant hybrid scaffolds, and only the second synthesis of these molecules overall. The driving force for the present method is a simple and inexpensive, green, iodine-[bmim][BF_4_] synergistic catalytic system cum reaction medium under ultrasound irradiation. The disclosed protocol proceeds at ambient temperature, under ligand-, metal-, and base-free conditions, is operationally simple, atom economic and the ionic liquid reaction media can be recycled, further enhancing its green credentials. The synthesized molecules exhibited good *in vitro* anti-proliferative activity against different cell lines cancer cell lines in an IC_50_ range of 5.45–19.10 µM. Further, the evaluated molecules were found to be non-toxic against normal human embryonic kidney (HEK-293) cell line. Compound 7 exhibited the best anti-cancer activity with IC_50_ values of 6.60, 5.45, 7.85, 11.21, 12.24, 10.12, and 11.32 µM against MCF-7, MDA-MB-231, HeLa, SKOV-3, A549, HCT-116 and DLD-1 cell lines, respectively, and can be considered as a new anti-cancer lead molecule.

## Data Availability

The original contributions presented in the study are included in the article/Supplementary Material, further inquiries can be directed to the corresponding authors.
