# Supplementary material for: An ultrasound assisted, ionic liquid-molecular iodine synergy driven efficient green synthesis of pyrrolobenzodiazepine-triazole hybrids as potential anticancer agents
Source: Front Pharmacol. 2023 May 5;14:1168566. doi: 10.3389/fphar.2023.1168566 (PMC10196072; doi:10.3389/fphar.2023.1168566)

Supplementary Material

An Ultrasound Assisted, Ionic Liquid-Molecular Iodine Synergy Driven Efficient Green Synthesis of Pyrrolobenzodiazepine-Triazole Hybrids as Potential Anticancer Agents

**Mohammad Saquib^1^, Shakir Ahamad^2^, Mohammad Faheem Khan^3^, Mohammad Imran Khan^4,5^* and Mohd. Kamil Hussain^6,7^***

^1^Department of Chemistry, University of Allahabad, Prayagraj (Allahabad), 211002, UP, India;

^2^Department of Chemistry, Aligarh Muslim University, Aligarh, 202002, UP, India

^3^Department of Biotechnology,Era’s Lucknow Medical College, Era University, Lucknow, 226003,U.P, India

^4^Department of Biochemistry, Faculty of Science, King Abdulaziz University, Jeddah, KSA

^5^ Centre of Artificial Intelligence in Precision Medicine, King Abdulaziz University, Jeddah, 21589, KSA

^6^Department of Chemistry, Govt. Raza P.G. College, Rampur, 244901,UP, India

^7^M.J.P Rohilkahand University, Bareilly, U.P. 243006, India

***Correspondence:**Corresponding Authors

Mohammad Imran Khan:[mikhan@kau.edu.sa](mailto:mikhan@kau.edu.sa)

Mohd. Kamil Hussain: [mkhcdri@gmail.com](mailto:mkhcdri@gmail.com)

**Content:**

1. General experimental information ………………………………………….1

2. General Procedure……………………………………………………………2

3. Characterization data for compounds **6-18………………………………**2 – 6

4. Copies of ^1^H and ^13^C NMR spectra for compounds **6-18………………**7 –19

**1. General experimental information:**

Unless otherwise specified, all reactions were carried out under an air atmosphere in an oven-dried round-bottom flask. The reactions were monitored by TLC visualized by UV (254 nm) and/or with iodine. Flash chromatography was performed on 100-200 mesh silica gel using the gradient system ethyl acetate-hexane (0-50%). NMR data were recorded at Bruker AV 300 MHz in CDCl_3_ using as internal standards the residual CHCl_3_ signal for ^1^H NMR (𝛿 = 7.26 ppm) and the deuterated solvent signal for ^13^C NMR (𝛿 = 77.16 ppm). Coupling constants are given in Hertz (Hz), and the classical abbreviations are used to describe the signal multiplicities. Melting points were measured with a Büchi B-540 apparatus and were uncorrected. All commercially available reagents were used as received.

**2. General procedure for the synthesis of Pyrrolobenzodiazepine-Fused Triazoles**.

To a mixture of propargyl alcohol (2.5 mmol) and 1-(2-azidoaryl)-1H-pyrroles (2 mmol) in [bmim][BF4] (4 ml) in a round bottom flask was added a catalytic amount of iodine (10 mol%) at room temperature and the reaction temperature was allowed to rise to 40 ^o^C. The reaction was stirred at this temperature for the next 1 hour and then it was subjected to ultrasound irradiation till completion of the reaction. The reaction mixture was now quenched with addition of water and extracted thrice with 10 ml of ethyl acetate. The combined organic extracts were treated with an aqueous solution of Na_2_S_2_O_3_ (1 M), washed with saturated solution of NaHCO_3_ and dried *in vacuo* to afford the crude product mixture, which was purified through column chromatography (EtOAc/hexane) to afford pure compound. The ionic liquid residue was washed with hexane and dried in vacuum to recover the [bmim][BF4] which was used in the next cycle.

**3. Characterization data for compounds 6-18**

**8-(4-methoxyphenyl)-9,9-dimethyl-9*H*-benzo[*b*]pyrrolo[1,2-*g*][1,2,3]triazolo[1,5-*d*][1,4]diazepine (6).**

Following the general procedure, treatment of aryl azide **1a** (368 mg, 2.0 mmol) with propargyl alcohol **2a** (475 mg, 2.5 mmol) and molecular iodine (10 mol%) in 4 ml of [bmim][BF4],followed by column chromatography afforded the product **6** as a colorless solid (321 mg, 90%). **R_f_** (Ethyl acetate/Hexane: 10/90) = 0.38. **^13^C NMR** (100 MHz, 𝛿 ppm/CDCl_3_): 159.8, 143.0, 140.5, 140.1, 132.3, 132.2, 132.2, 130.2, 129.7, 126.9, 125.3, 125.1, 123.4, 122.07, 113.4, 113.4, 110.3, 106.3, 55.4, 33.9, 27.6, 26.5. **^1^H NMR** (300 MHz, 𝛿 ppm/CDCl_3_): 8.09 (d, *J* = 8.1 Hz, 1H), 7.55 (d, *J* = 4.5 Hz, 1H) -7.50-7.44 (m, 2H), 7.33 (d, *J* = 7.5 Hz, 2H),7.03 (s, 1H), 6.94 (d, *J* = 8.1 Hz, 2H), 6.28 (t, *J* = 3.0 Hz, 1H), 6.04 (s, 1H), 3.86 (s, 3H), 1.55 (s, 3H), 1.28 (s, 3H).

**9,9-dimethyl-8-phenyl-9*H*-benzo[*b*]pyrrolo[1,2-*g*][1,2,3]triazolo[1,5-*d*][1,4]diazepine (7).**

 Following the general procedure, treatment of aryl azide **1a** (368 mg, 2.0 mmol) with propargyl alcohol **2b** (400 mg, 2.5 mmol) and molecular iodine (10 mol%) in 4 ml of [bmim][BF4], followed by column chromatography afforded the product **7** as a colorless solid (281 mg, 86%). **R_f_** (Ethyl acetate/Hexane: 10/90) = 0.50. **^13^C NMR** (100 MHz, 𝛿 ppm/CDCl_3_): 143.4, 140.6, 140.1, 133.00, 133.0, 132.3, 131.1, 130.1, 129.8, 128.6, 128.0, 128.0, 126.9, 125.3, 123.4, 122.1, 110.3, 106.4, 33.9, 27.7, 26.5. **^1^H NMR** (300 MHz, 𝛿 ppm/CDCl_3_): 8.11 (d, *J* = 5.7 Hz, 1H), 7.57-7.56 (m, 2H), 7.53-7.46 (m, 1H), 7.45-7.41 (m, 5H), 7.04-7.03 (m, 1H), 6.30-6.28 (m, 1H), 6.05-6.04 (m, 1H), 1.55 (s, 3H), 1.30 (s, 3H).

**1,3,9,9-tetramethyl-8-phenyl-9*H*-benzo[*b*]pyrrolo[1,2-*g*][1,2,3]triazolo[1,5-*d*][1,4]diazepine (8).**

Following the general procedure, treatment of aryl azide **1b** (424 mg, 2.0 mmol) with propargyl alcohol **2b** (400 mg, 2.5 mmol) and molecular iodine (10 mol%) in 4 ml of [bmim][BF4], followed by column chromatography afforded the product **8** as a colorless solid (301 mg, 85%). **R_f_** (Ethyl acetate/Hexane: 10/90) = 0.48. **^13^C NMR** (100 MHz, 𝛿 ppm/CDCl_3_): 143.1, 141.4, 140.7, 137.4, 133.0, 132.9, 132.3, 132.0, 131.0, 131.0, 129.4, 128.5, 127.9, 127.9, 124.4, 123.8, 108.3, 104.8, 34.1, 27.7, 25.4, 21.0, 20.0. **^1^H NMR** (300 MHz, 𝛿 ppm/CDCl_3_): 7.67 (d, *J* = 1.5 Hz, 1H), 7.42-7.38 (m, 5H), 7.25 (s, 1H), 6.81-6.80 (m, 1H), 6.19-6.17 (m, 1H), 5.97-5.96 (m, 1H), 2.46 (s, 3H), 2.42 (s, 3H), 1.52 (s, 3H), 1.25 (s, 3H).

**1-(4-(9,9-dimethyl-9*H*-benzo[*b*]pyrrolo[1,2-*g*][1,2,3]triazolo[1,5-*d*][1,4]diazepin-8-**

**yl)phenyl)ethanone (9).**

Following the general procedure, treatment of aryl azide **1a** (368 mg, 2.0 mmol) with propargyl alcohol **2c** (505 mg, 2.5 mmol) and molecular iodine (10 mol%) in 4 ml of [bmim][BF4], followed by column chromatography afforded the product **9** as a colorless solid (302 mg, 82%). **R_f_** (Ethyl acetate/Hexane: 10/90) = 0.40. **^13^C NMR** (100 MHz, 𝛿 ppm/CDCl_3_): 197.8, 142.3, 140.8, 139.7, 137.8, 137.1, 132.3, 131.4, 131.4, 130.0, 129.9, 128.0, 127.0, 127.0, 125.3, 123.54, 122.2, 110.4, 106.5, 33.9, 27.8, 26.8, 26.4. **^1^H NMR** (300 MHz, 𝛿 ppm/CDCl_3_): 8.10 (d, *J* = 5.1 Hz, 1H), 8.02 (dd, *J* = 6.1, 1.5 Hz, 2H), 7.59-7.50 (m, 3H), 7.49-7.47 (m, 2H), 7.05-7.04 (m, 1H), 6.29 (t, *J* = 2.2 Hz, 1H), 6.05-6.04 (m, 1H), 2.66 (s, 3H),1.55 (s, 3H), 1.31 (s, 3H).

**1-(4-(1,3,9,9-tetramethyl-9*H*-benzo[*b*]pyrrolo[1,2-*g*][1,2,3]triazolo[1,5-*d*][1,4]diazepin-8-**

**yl)phenyl)ethanone (10).**

Following the general procedure, treatment of aryl azide **1b** (424 mg, 2.0 mmol) with propargyl alcohol **2c** (505 mg, 2.5 mmol) and molecular iodine (10 mol%) in 4 ml of [bmim][BF4] at room temperature followed by column chromatography afforded the product **10** as a colorless solid (317 mg, 80%). **R_f_** (Ethyl acetate/Hexane: 10/90) = 0.35. **^13^C NMR** (100 MHz, 𝛿 ppm/CDCl_3_): 197.89, 142.08, 141.81, 140.38, 138.00, 137.60, 137.07, 133.17, 132.39, 131.84, 131.43, 131.43, 129.39, 127.97, 127.97, 124.50, 123.80, 108.43, 104.95, 34.18, 27.91, 26.84, 25.32, 21.01, 19.99. **^1^H NMR** (300 MHz, 𝛿 ppm/CDCl_3_): 8.01 (dd, *J* = 4.8, 1.5 Hz, 2H), 7.66 (t, *J* = 0.9 Hz, 1H), 7.52 (dd, *J* = 4.8, 1.2 Hz, 2H), 7.26 (s, 1H), 6.82-6.81 (m, 1H), 6.19 (t, *J* = 1.2 Hz, 1H), 5.97-5.96 (m, 1H), 2.66 (s, 3H), 2.46 (s,3H), 2.43 (s, 3H),1.52 (s, 3H), 1.26 (s, 3H).

**8-(4-methoxyphenyl)-1,3,9,9-tetramethyl-9*H*-benzo[*b*]pyrrolo[1,2-*g*][1,2,3]triazolo[1,5-*d*]**

**[1,4]diazepine (11)**

Following the general procedure, treatment of aryl azide **1b** (424 mg, 2.0 mmol) with propargyl alcohol **2a** (475 mg, 2.5 mmol) and molecular iodine (10 mol%) in 4 ml of [bmim][BF4], followed by column chromatography afforded the product **11** as a colorless solid (338 mg, 88%). **R_f_** (Ethyl acetate/Hexane: 10/90) = 0.44. **^13^C NMR** (100 MHz, 𝛿 ppm/CDCl_3_): 159.8, 142.8, 141.4, 140.7, 137.4, 132.9, 132.2, 132.2, 132.2, 132.0, 129.4, 125.2, 124.3, 123.8, 113.4, 113.4, 108.3, 104.8, 55.4, 34.1, 27.7, 25.4, 21.0, 20.0. **^1^H NMR** (300 MHz, 𝛿 ppm/CDCl_3_): 7.66 (d, *J* = 1.5 Hz, 1H), 7.32-7.29 (m, 2H), 7.24-7.23 (m, 1H), 6.95-6.92 (m, 2H), 6.81-6.80 (m, 1H), 6.17 (t, *J* = 1.2 Hz, 1H), 5.97-5.96 (m, 1H), 3.86 (s, 3H), 2.45 (s, 3H), 2.42 (s, 3H), 1.53 (s, 3H), 1.24 (s, 3H).

**9-(4-chlorophenyl)-8-cyclopropyl-9H-benzo[b]pyrrolo[1,2-d][1,2,3]triazolo[5,1-g][1,4]diazepine (12).**

 Following the general procedure, treatment of aryl azide **1a** (368 mg, 2.0 mmol) with propargyl alcohol **3a** (705 mg, 2.5 mmol) and molecular iodine (10 mol%) in 4 ml of [bmim][BF4], followed by column chromatography afforded the product **12** as a colorless solid (321 mg, 86%). **R_f_** (Ethyl acetate/Hexane: 10/90) = 0.50. **^13^C NMR** (100 MHz, 𝛿 ppm/CDCl_3_): 145.9, 136.1, 135.8, 133.0, 132.4, 131.4, 129.6, 129.2, 128.8, 128.6, 128.4, 128.3, 127.0, 124.6, 123.6, 122.5, 111.0, 110.3, 36.6, 7.0, 6.9, 5.6. **^1^H NMR** (300 MHz, 𝛿 ppm/CDCl_3_): 7.81 (d, *J* = 7.2 Hz, 1H), 7.35-7.31 (m, 4H), 7.03-7.00 (m, 3H), 6.67 (d, *J* = 6.0 Hz, 2H), 6.38 (d, *J* = 1.8 Hz, 1H), 5.79 (s, 1H), 1.99-1.91 (m, 1H), 1.11-1.01 (m, 4H).

**9-(4-chlorophenyl)-8-cyclopropyl-1,3-dimethyl-9*H*-benzo[*b*]pyrrolo[1,2- *g*][1,2,3]triazolo[1,5-*d*][1,4]diazepine (13).**

 Following the general procedure, treatment of aryl azide **1b** (424 mg, 2.0 mmol) with propargyl alcohol **3a** (705 mg, 2.5 mmol) and molecular iodine (10 mol%) in 4 ml of [bmim][BF4], followed by column chromatography afforded the product **13** as a colorless solid (337 mg, 84%). **R_f_** (Ethyl acetate/Hexane: 10/90) = 0.46. **^13^C NMR** (100 MHz, 𝛿 ppm/CDCl_3_): 145.6, 137.56, 136.69, 136.31, 133.38, 132.85, 132.53, 131.20, 128.39, 128.39, 128.16, 128.03, 128.03, 127.61, 124.8, 122.6, 109.0, 108.6, 36.8, 20.9, 19.7, 6.8, 6.8, 5.5. **^1^H NMR** (300 MHz, 𝛿 ppm/CDCl_3_): 7.38 (s, 1H), 7.01 (d, *J* = 6.6 Hz, 2H), 6.94 (s, 1H), 6.79-6.74 (m, 3H), 6.30-6.25 (m, 2H), 5.73 (s, 1H), 2.27 (s, 3H), 2.22 (s, 3H), 1.94-1.88 (m, 1H), 1.04-1.00 (m, 4H).

**8-cyclopropyl-9-(3,4,5-trimethoxyphenyl)-9*H*-benzo[*b*]pyrrolo[1,2-*g*][1,2,3]triazolo[1,5-**

***d*][1,4]diazepine (14).**

Following the general procedure, treatment of aryl azide **1** (368 mg, 2.0 mmol) with propargyl alcohol **3b** (845 mg, 2.5 mmol) and molecular iodine (10 mol%) in 4 ml of [bmim][BF4], followed by column chromatography afforded the product **14** as a colorless solid (386 mg, 90%). **R_f_** (Ethyl acetate/Hexane: 10/90) = 0.20. **^13^C NMR** (100 MHz, 𝛿 ppm/CDCl_3_): 153.1, 153.1, 145.6, 145.6, 137.0, 136.2, 133.3, 132.8, 131.6, 129.5, 129.4, 126.9, 124.7, 123.7, 122.3, 111.0, 110.2, 104.5, 77.5, 60.8, 56.1, 37.2, 7.0, 6.7, 5.6. **^1^H NMR** (300 MHz, 𝛿 ppm/CDCl_3_): 7.85 (d, *J* = 5.7 Hz, 1H), 7.37-7.34 (m, 2H), 7.29-7.27 (m, 1H), 7.03-7.02 (m, 1H), 6.37-6.36 (d, *J* = 2.7 Hz, 2H), 5.95 (s, 2H), 5.79 (s, 1H), 3.68 (s, 3H), 3.56 (s, 6H), 2.04-1.95 (m, 1H), 1.15-.1.03 (m, 4H).

**9-(4-methoxyphenyl)-8-phenyl-9H-benzo[*b*]pyrrolo[1,2-g][1,2,3]triazolo[1,5-**

***d*][1,4]diazepine**(**15**).

Following the general procedure, treatment of aryl azide **1a** (368 mg, 2.0 mmol) with propargyl alcohol **4** (695 mg, 2.5 mmol) and molecular iodine (10 mol%) in 4 ml of [bmim][BF4], followed by column chromatography afforded the product **15** as a colorless solid (372 mg, 92%). **R_f_** (Ethyl acetate/Hexane: 10/90) = 0.47. **^13^C NMR** (75 MHz, 𝛿 ppm/CDCl_3_): 158.5, 144.9, 135.8, 133.0, 131.7, 130.6, 129.7, 129.7, 129.3, 129.1, 129.1, 128.5, 128.0, 128.0, 127.8, 127.8, 126.8, 124.8, 123.5, 122.2, 113.9, 113.9, 111.0, 110.3, 55.2, 37.0. **^1^H NMR** (300 MHz, 𝛿 ppm/CDCl_3_): 7.90 (d, *J* = 7.8 Hz, 1H), 9.6 (d, *J* = 7.2 Hz, 2H), 7.52-7.42 (m, 3H), 7.35-7.33 (m, 2H), 7.29-7.25 (m, 1H), 7.05-7.03 (m, 1H), 6.69 (d, *J* = 8.4 Hz, 2H), 6.58 (d, *J* = 6.6 Hz, 2H), 6.37-6.34 (m, 2H), 5.97 (s, 1H), 3.64 (s, 3H).

**9-(4-methoxyphenyl)-1,3-dimethyl-8-phenyl-9*H*-benzo[*b*]pyrrolo[1,2-*g*][1,2,3]triazolo[1,5-**

***d*][1,4]diazepine (16).**

Following the general procedure, treatment of aryl azide **1b** (424 mg, 2.0 mmol) with propargyl alcohol **4** (695 mg, 2.5 mmol) and molecular iodine (10 mol%) in 4 ml of [bmim][BF4], followed by column chromatography afforded the product **16** as a colorless solid (385 mg, 89%). **R_f_** (Ethyl acetate/Hexane: 10/90) = 0.45. **^13^C NMR** (100 MHz, 𝛿 ppm/CDCl_3_): 158.4, 144.7, 137.2, 136.6, 134.0, 132.6, 132.4, 131.2, 130.7, 129.2, 129.2, 129.1, 128.4, 127.8, 127.8, 127.6, 127.6, 124.5, 122.7, 113.7, 113.7, 109.0, 108.5, 55.2, 37.2, 20.9, 19.7. **^1^H NMR** (300 MHz, 𝛿 ppm/CDCl_3_): 7.73 (d, *J* = 5.1 Hz, 2H), 7.50-7.46 (m, 3H), 7.43-7.39 (m, 1H), 6.95-6.94 (m, 1H), 6.81-6.75 (m, 3H), 6.58 (d, *J* = 6.6 Hz, 2H), 6.25 (d, *J* = 1.8 Hz, 2H), 5.91 (s, 1H), 3.67 (s, 3H), 2.29 (s, 3H), 2.23 (s, 3H).

**9-(furan-2-yl)-8-(4-methoxyphenyl)-9*H*-benzo[*b*]pyrrolo[1,2-*g*][1,2,3]triazolo[1,5-**

***d*][1,4]diazepine( 17).**

Following the general procedure, treatment of aryl azide **1a** (368 mg, 2.0 mmol) with propargyl alcohol **5** (595 mg, 2.5 mmol) and molecular iodine (10 mol%) in 4 ml of [bmim][BF4], followed by column chromatography afforded the product **17** as a colorless solid (379 mg, 96%). **R_f_** (Ethyl acetate/Hexane: 10/90) = 0.38. **^13^C NMR** (75 MHz, 𝛿 ppm/CDCl_3_): 160.0, 149.8, 144.4, 142.4, 133.1, 131.6, 130.6, 129.8, 129.4, 129.4, 129.2, 127.0, 125.0, 123.6, 122.8, 122.5, 114.6, 114.62, 111.1, 110.3, 109.9, 107.8, 55.4, 33.4. **^1^H NMR** (300 MHz, 𝛿 ppm/CDCl_3_): 7.97 (d, *J* = 7.8 Hz, 1H), 7.71 (d, *J* = 7.5 Hz, 2H), 7.47 (d, *J* = 3.6 Hz, 2H) 7.45-7.34 (m, 1H), 7.15 (s, 1H), 7.07-7.04 (m, 3H), 6.35-627 (m, 2H), 6.03 (s, 1H), 5.93 (s, 1H), 5.57 (s, 1H), 3.87 (s, 3H)

**9-(furan-2-yl)-8-(4-methoxyphenyl)-1,3-dimethyl-9*H*-benzo[*b*]pyrrolo[1,2-**

***g*][1,2,3]triazolo[1,5-*d*][1,4]diazepine (18).**

 Following the general procedure, treatment of aryl azide **1b** (424 mg, 2.0 mmol) with propargyl alcohol **5** (595 mg, 2.5 mmol) and molecular iodine (10 mol%) in 4 ml of [bmim][BF4] followed by column chromatography afforded the product **18** as a colorless solid (401 mg, 95%). **R_f_** (Ethyl acetate/Hexane: 10/90) = 0.45. **^13^C NMR** (100 MHz, 𝛿 ppm/CDCl_3_): 159.9, 149.9, 144.2, 142.2, 137.5, 134.1, 132.7, 132.6, 131.5, 131.1, 129.4, 129.4, 128.4, 124.9, 123.1, 123.0, 114.5, 114.5, 110.4, 109.1, 108.2, 107.3, 55.4, 33.6, 20.9, 19.8. **^1^H NMR** (300 MHz, 𝛿 ppm/CDCl_3_): 7.69 (d, *J* = 9.6 Hz, 2H), 7.53 (s, 1H), 7.13-7.10 (m, 2H), 7.06-7.03 (m, 2H), 6.82-6.81(m, 1H), 6.23 (t, *J* = 5.7 Hz, 1H), 6.19-6.18 (m, 1H), 6.04-6.03 (m, 1H), 5.59(s, 1H), 5.29 (s, 1H), 3.87 (s, 3H), 2.36 (s, 3H), 2.34 (s, 3H).

**4. Copies of ^1^H and ^13^C NMR spectra for 6-18**


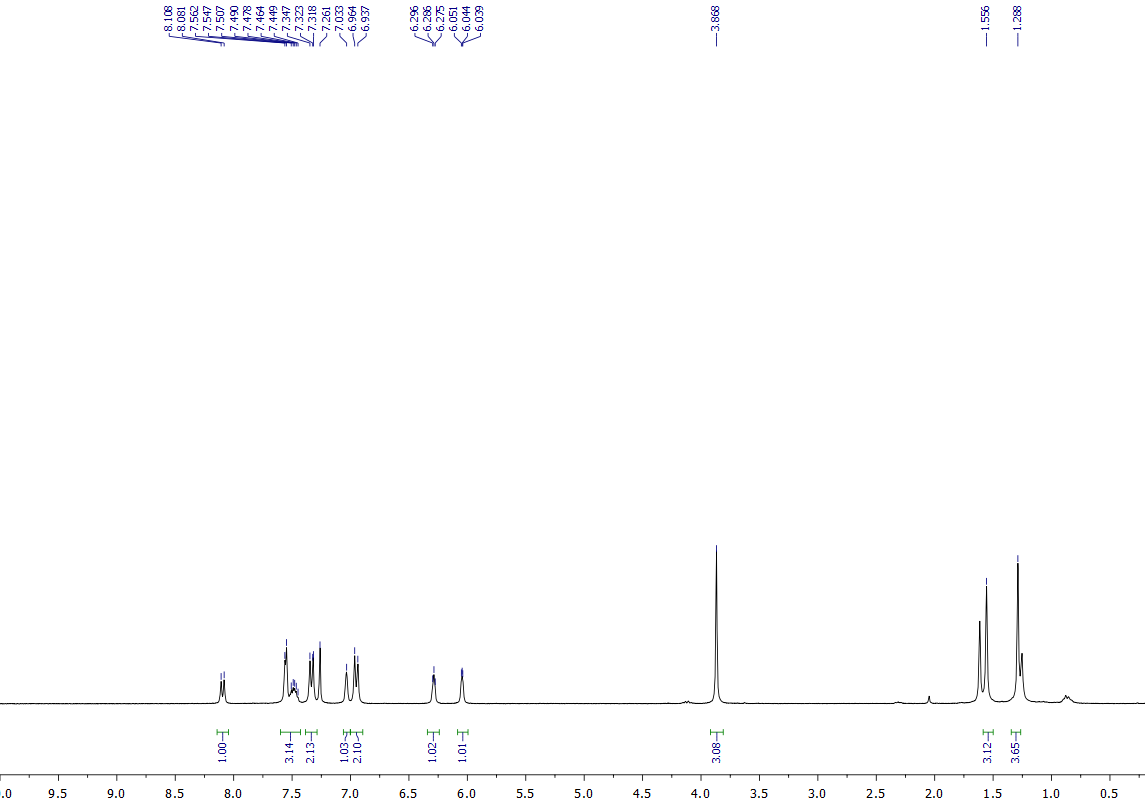


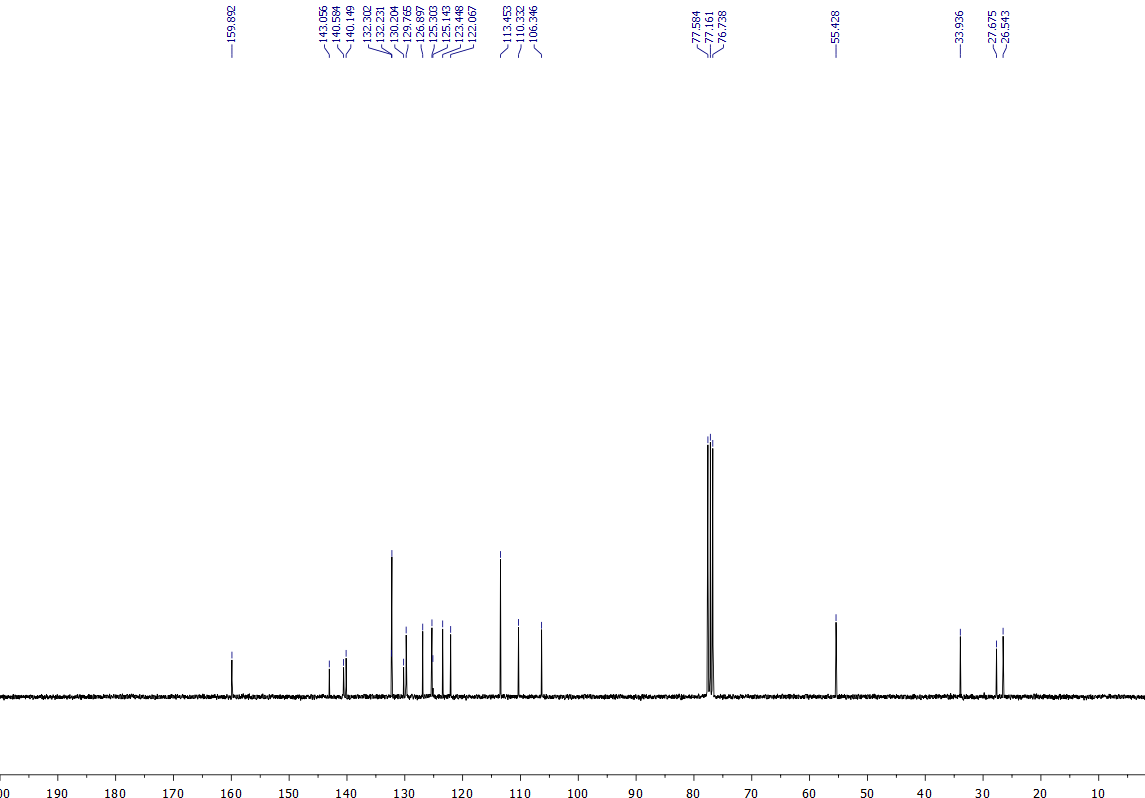


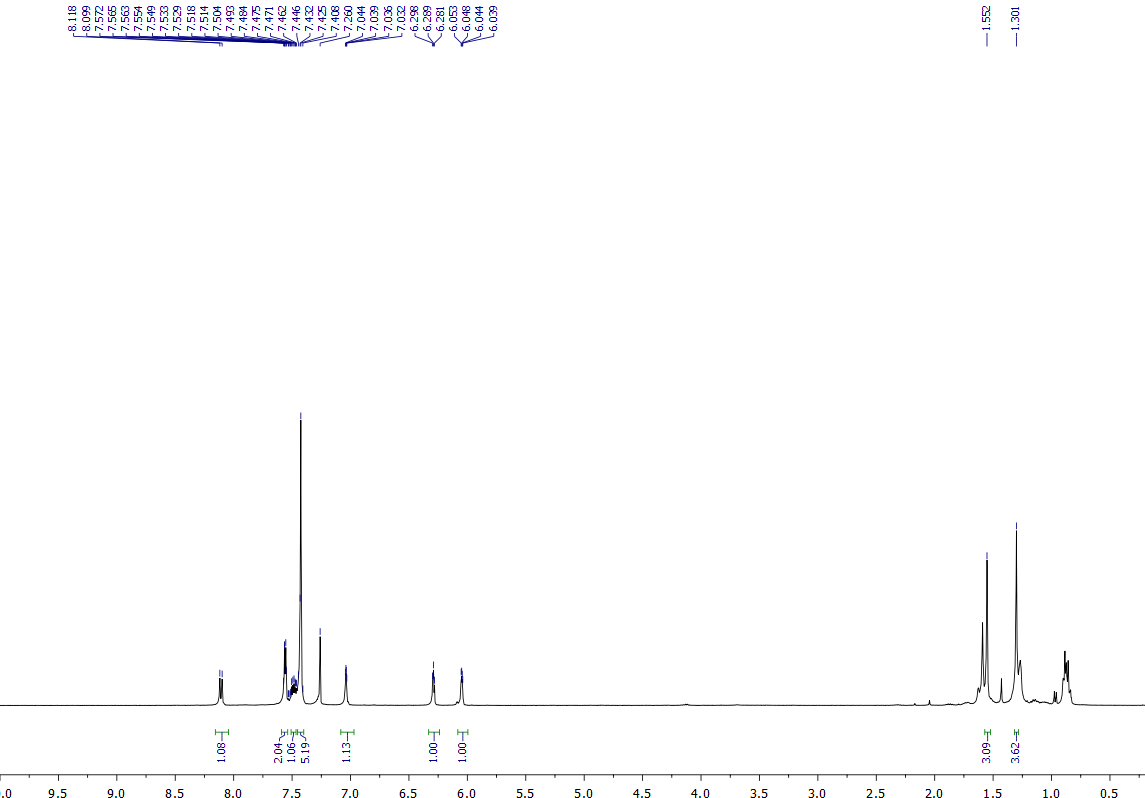

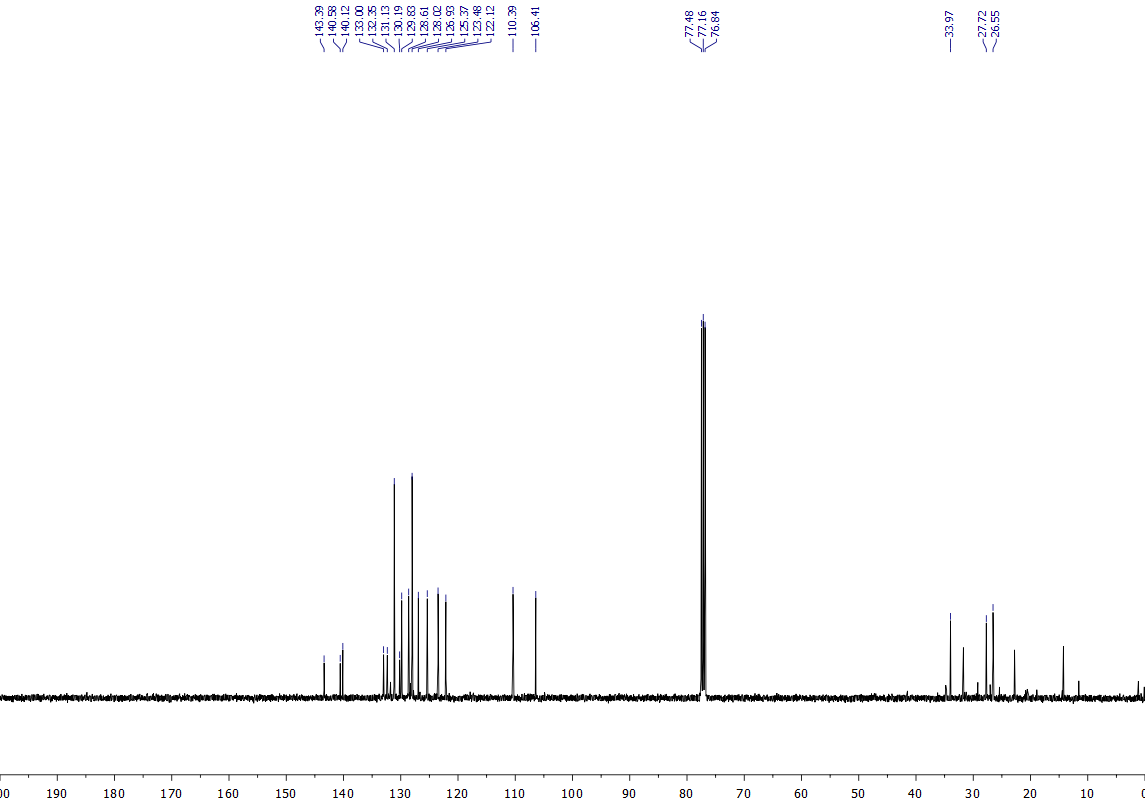


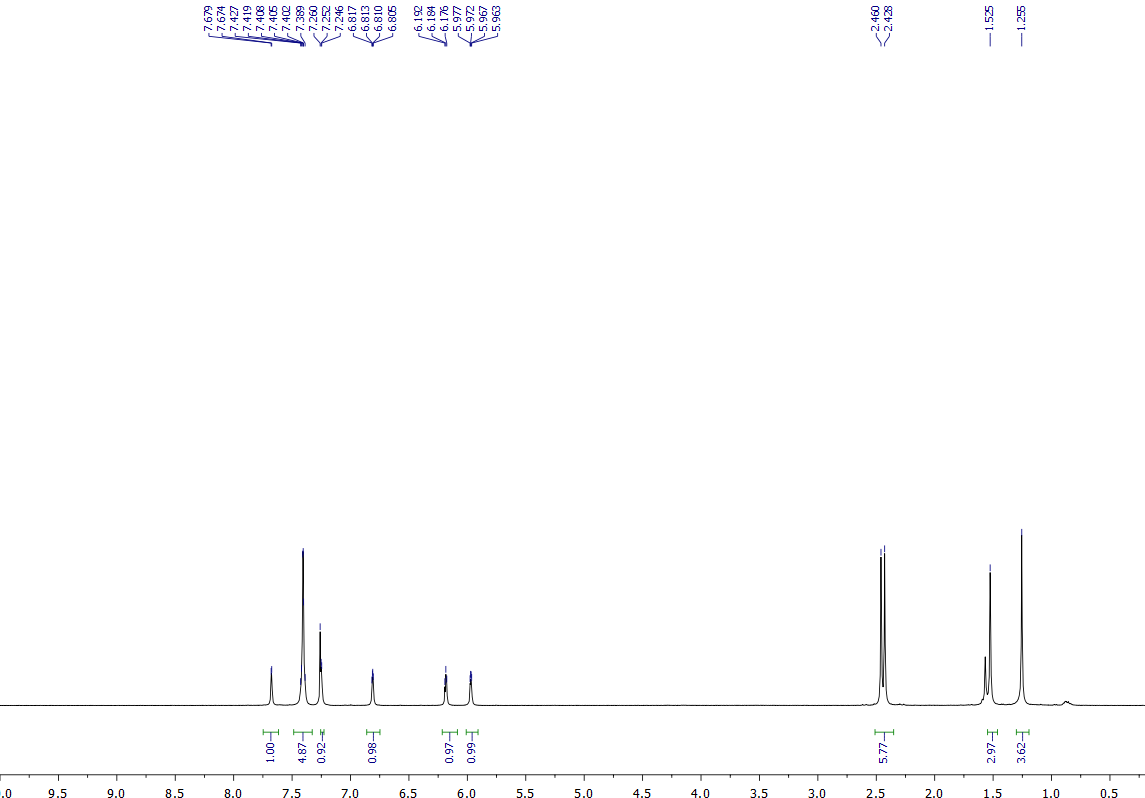

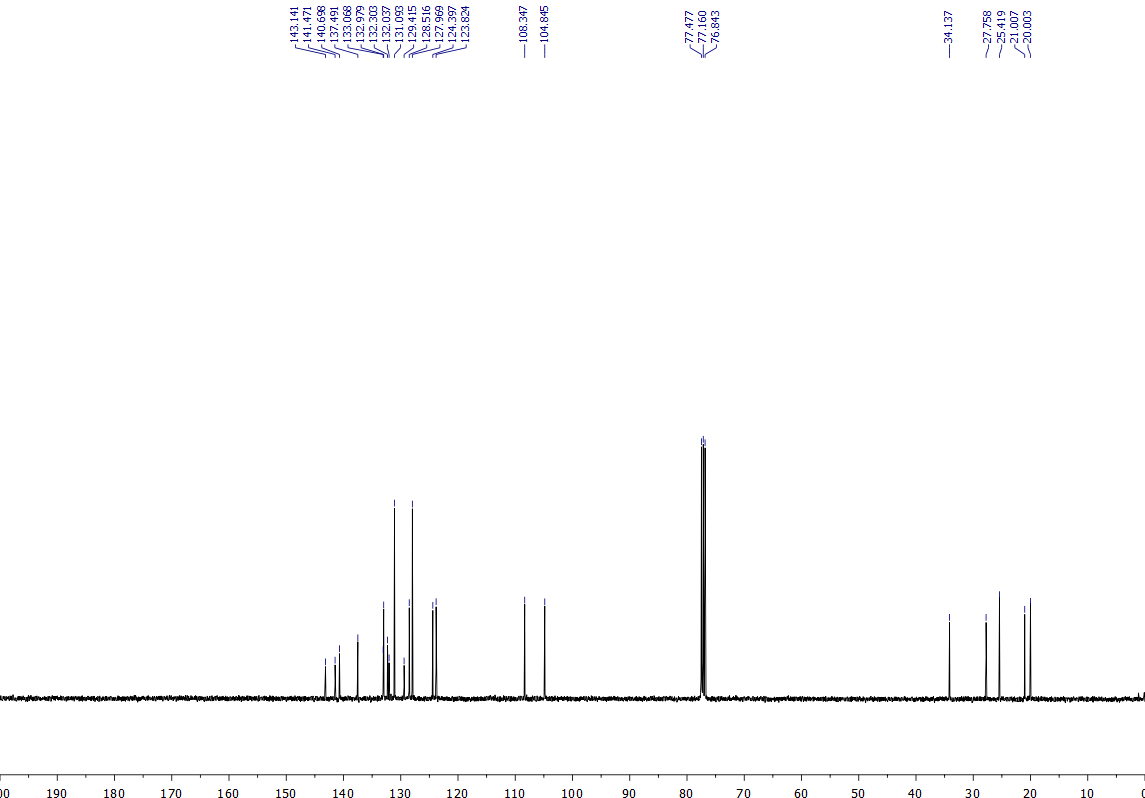


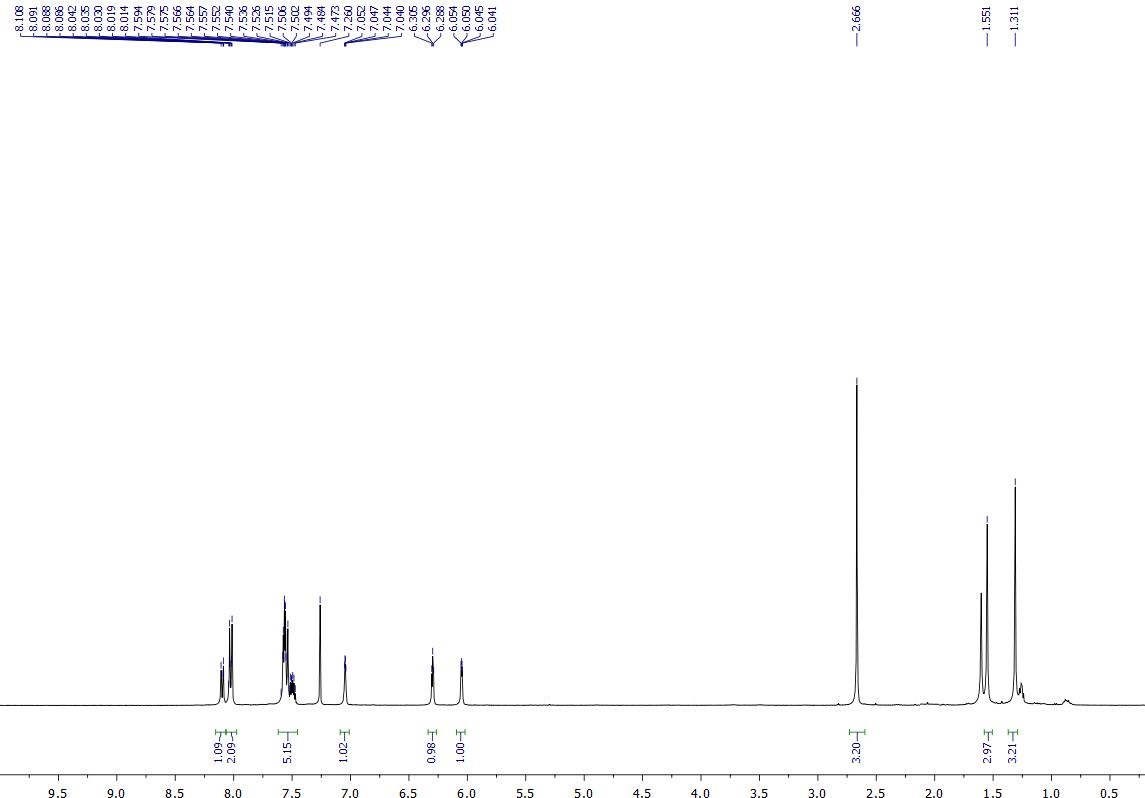


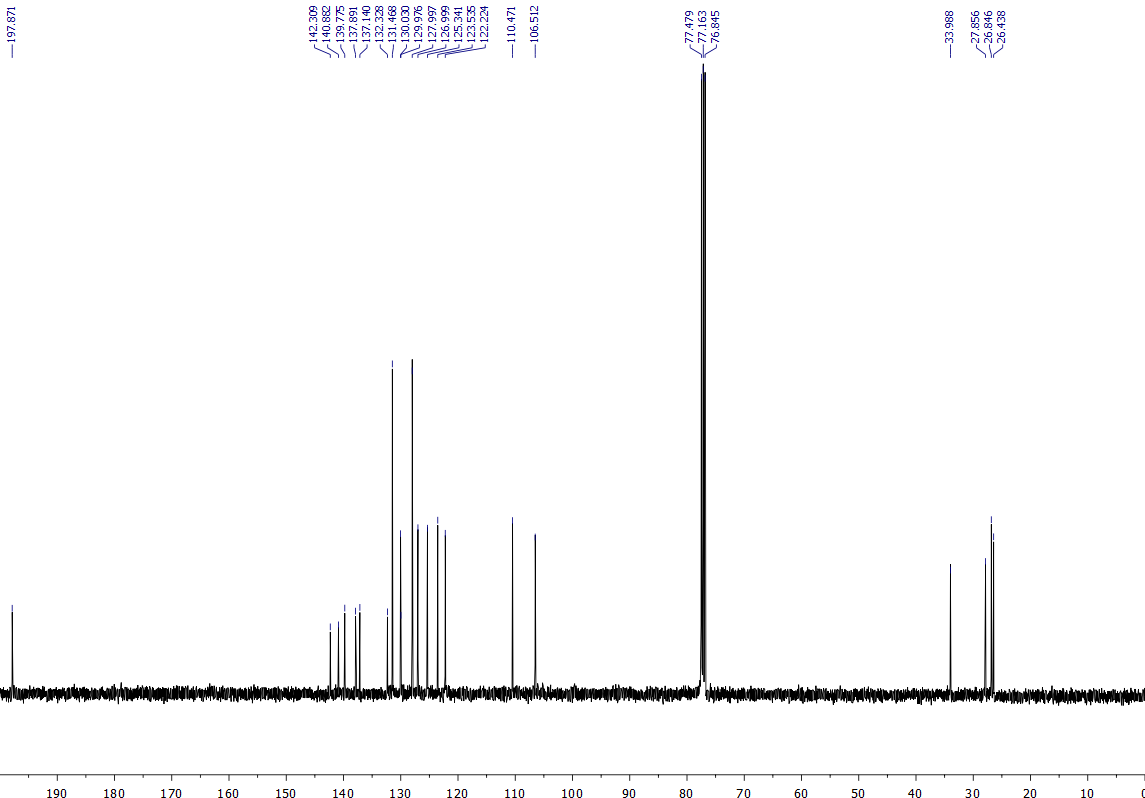


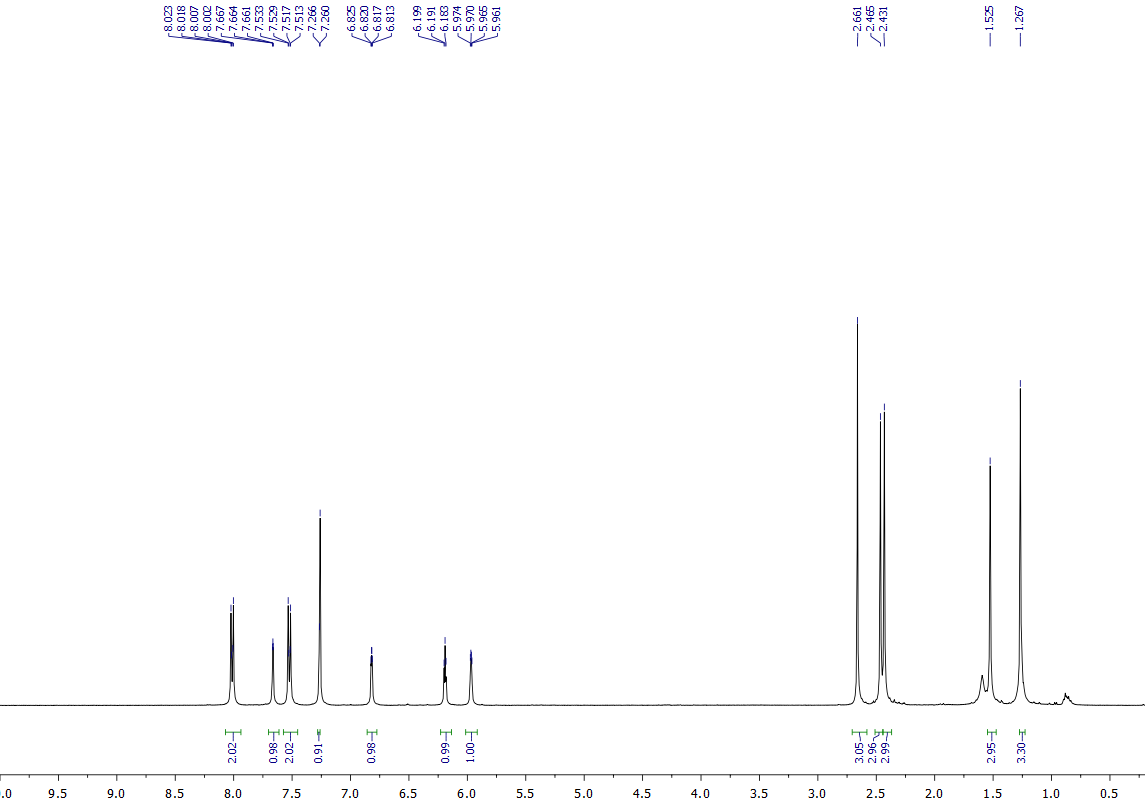


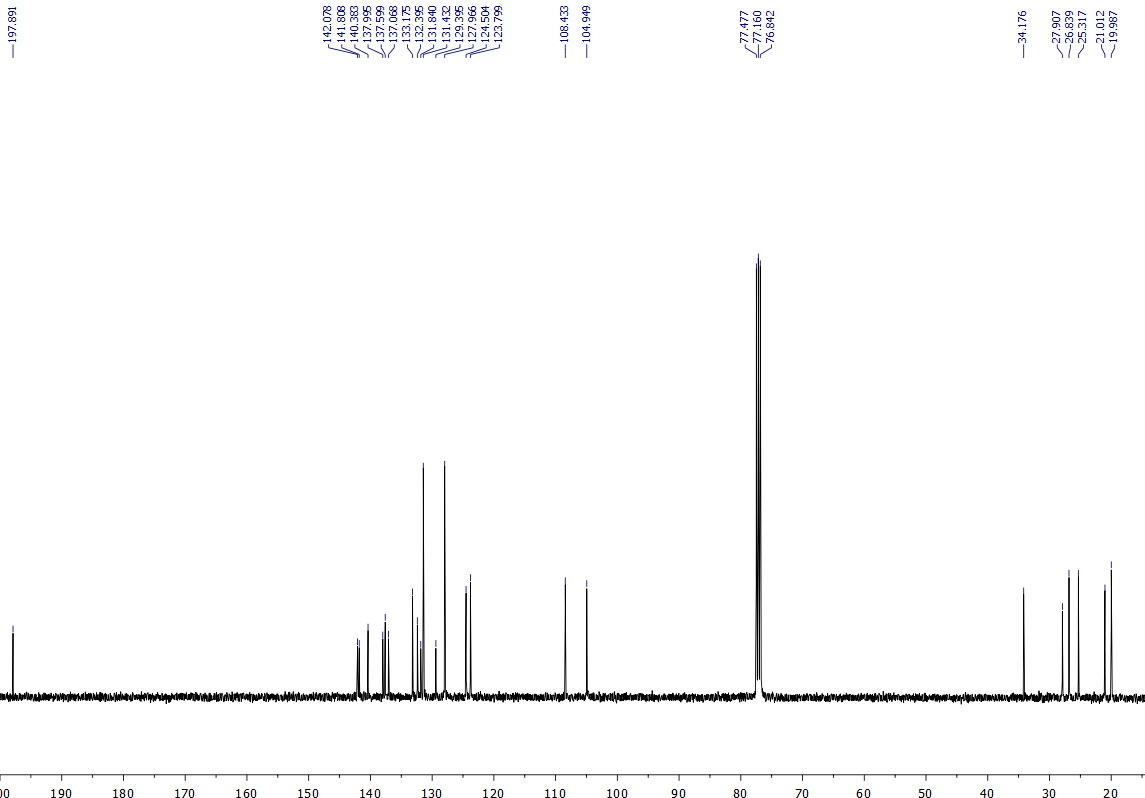


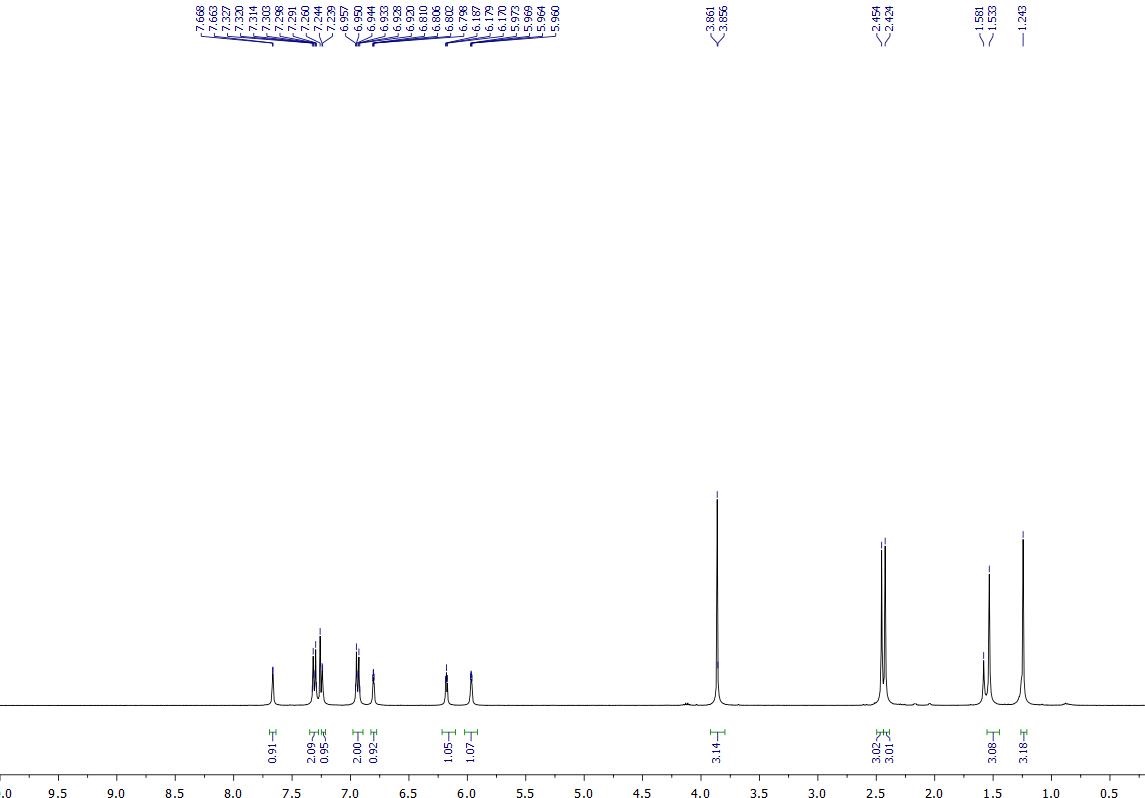


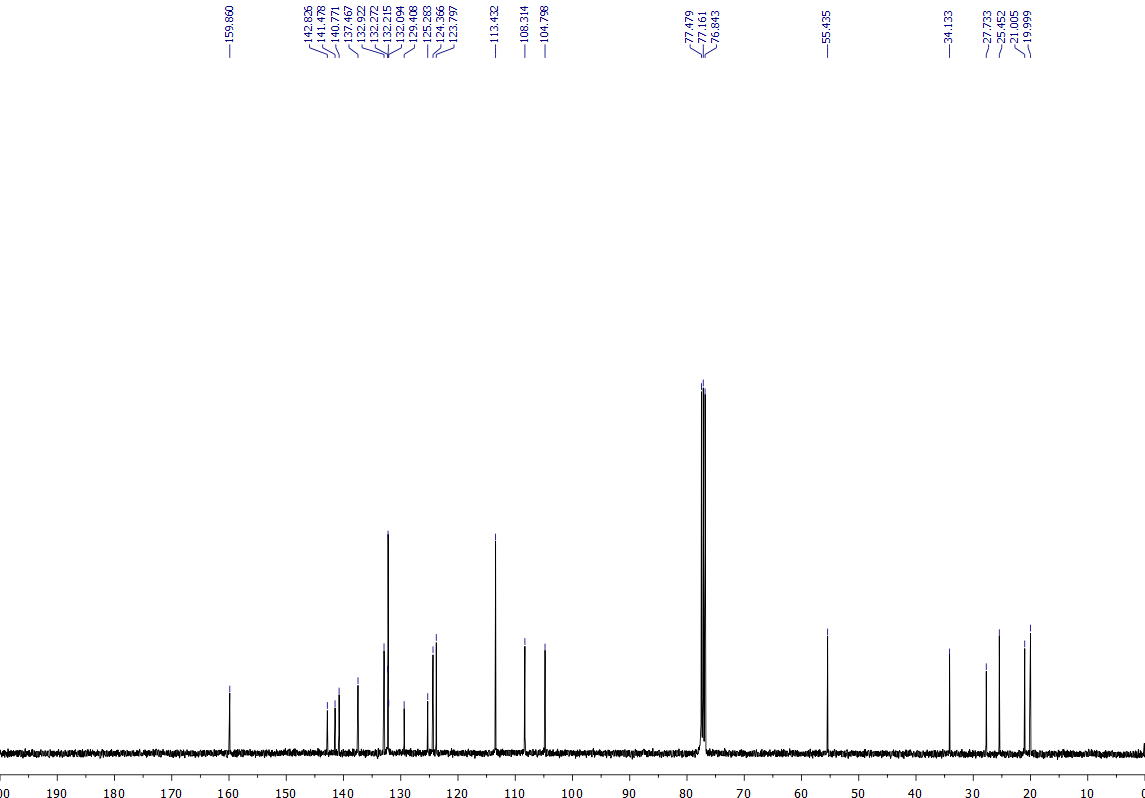


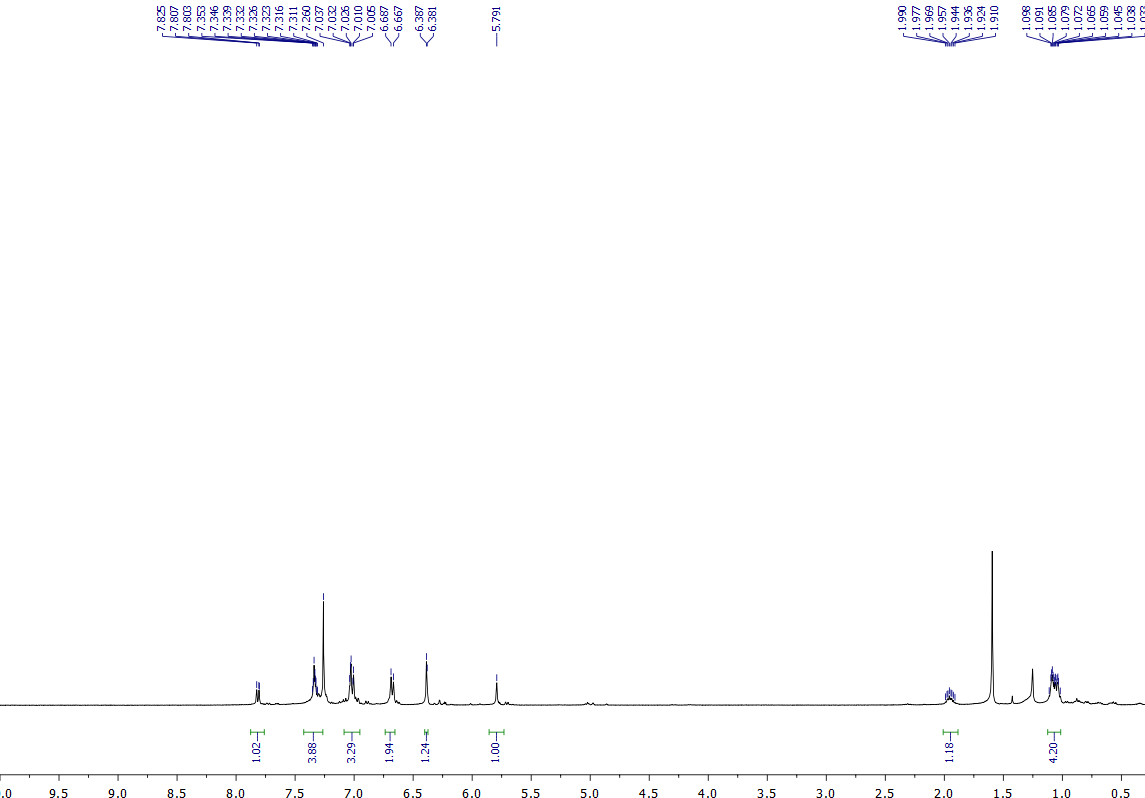

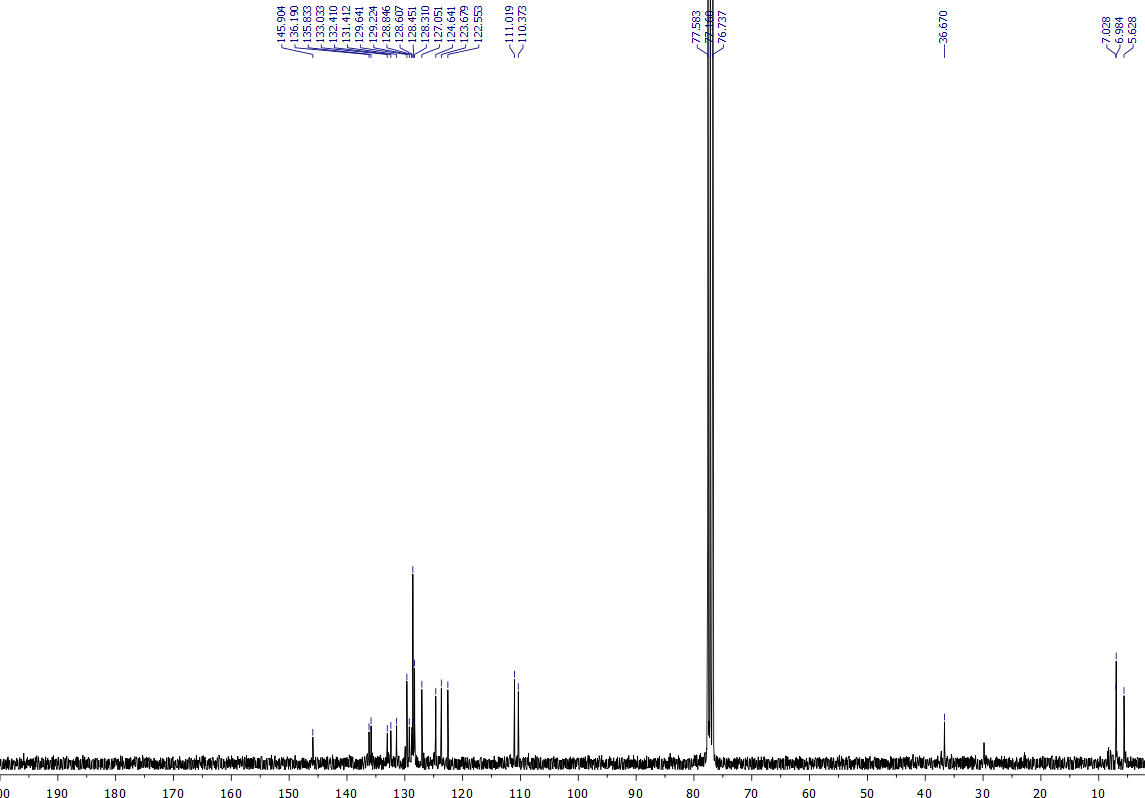


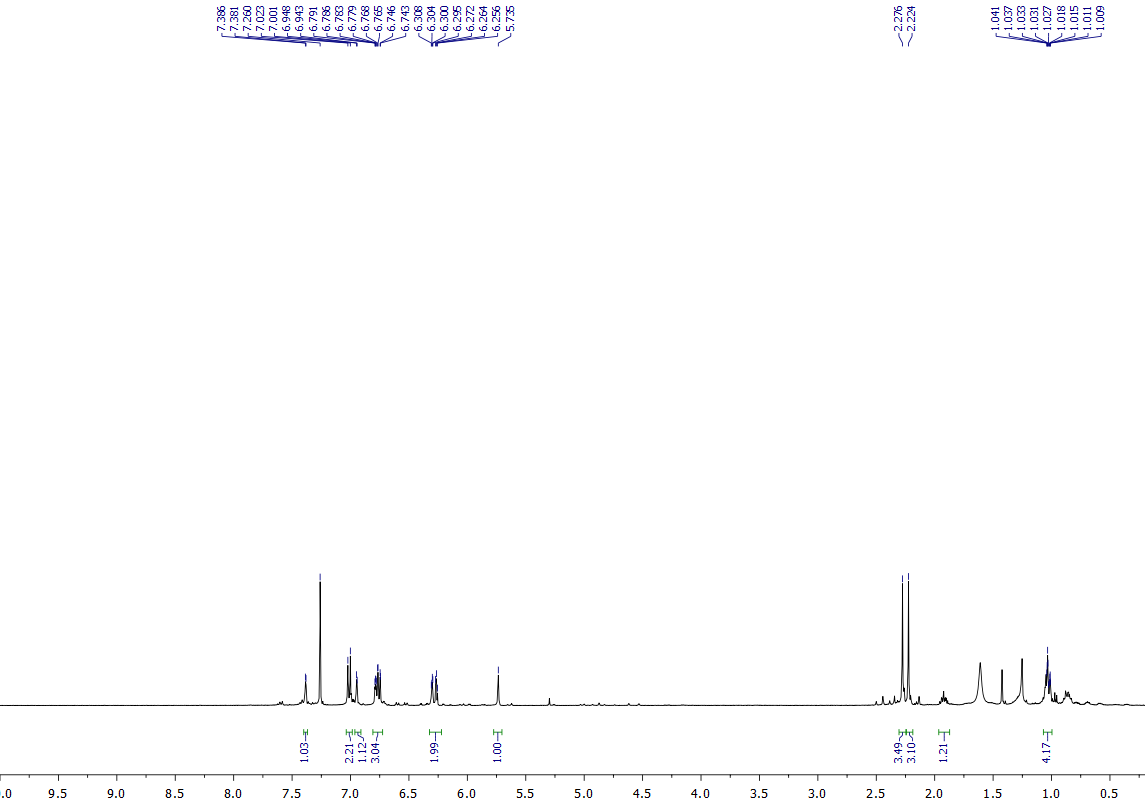

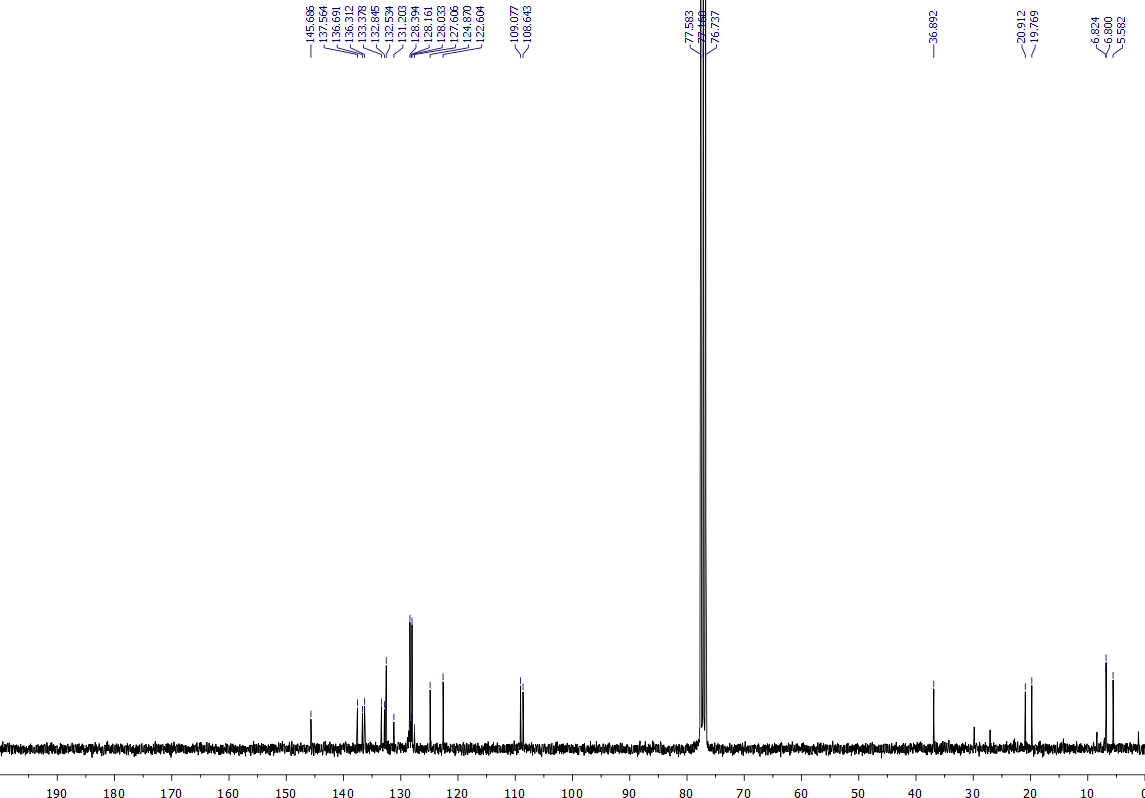


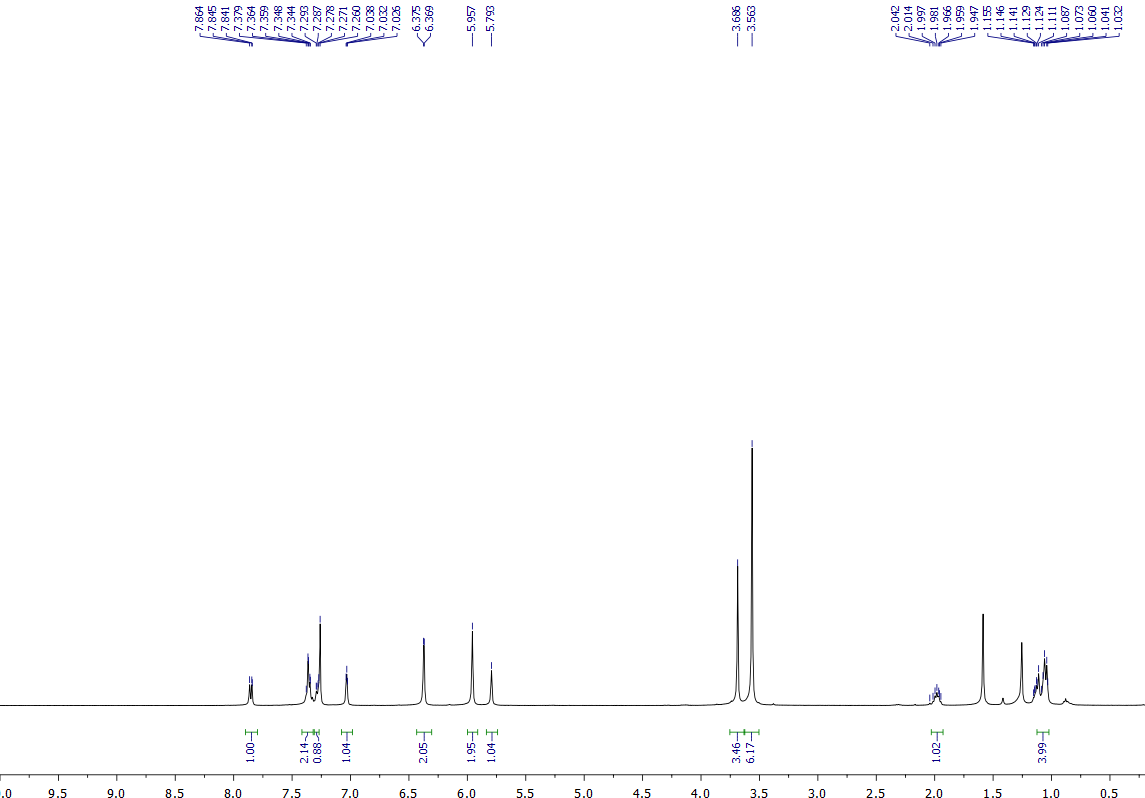

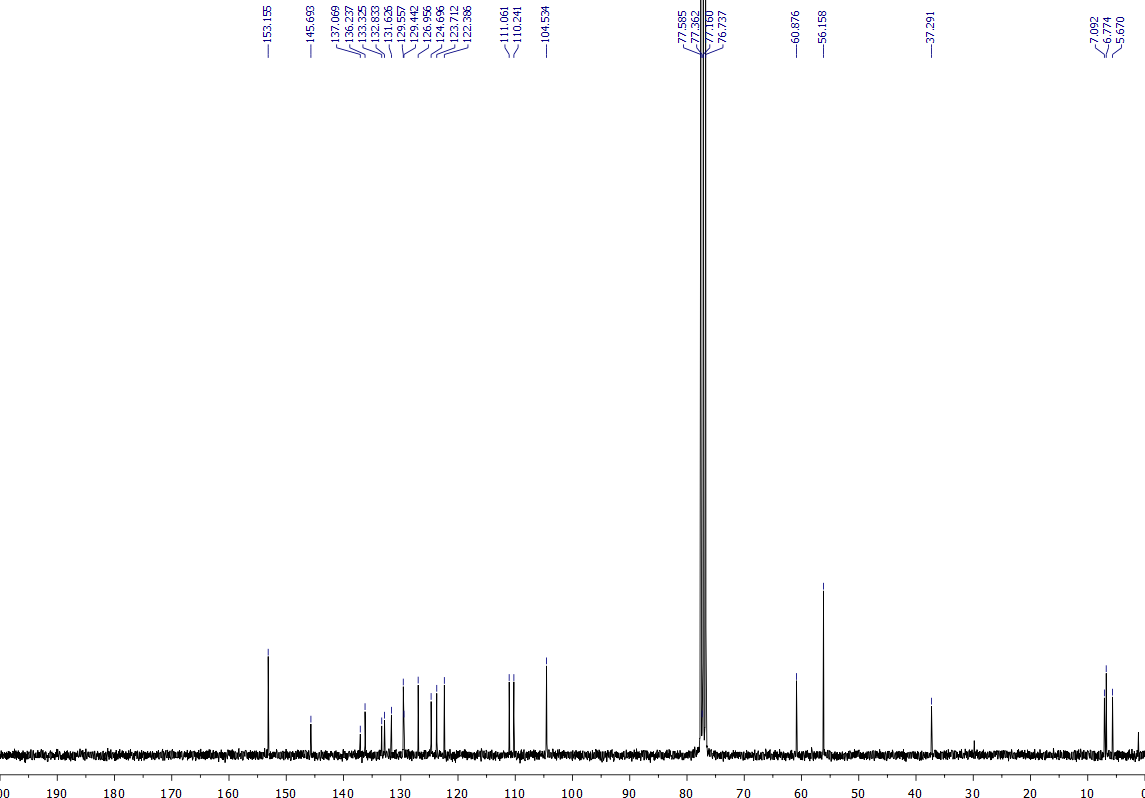


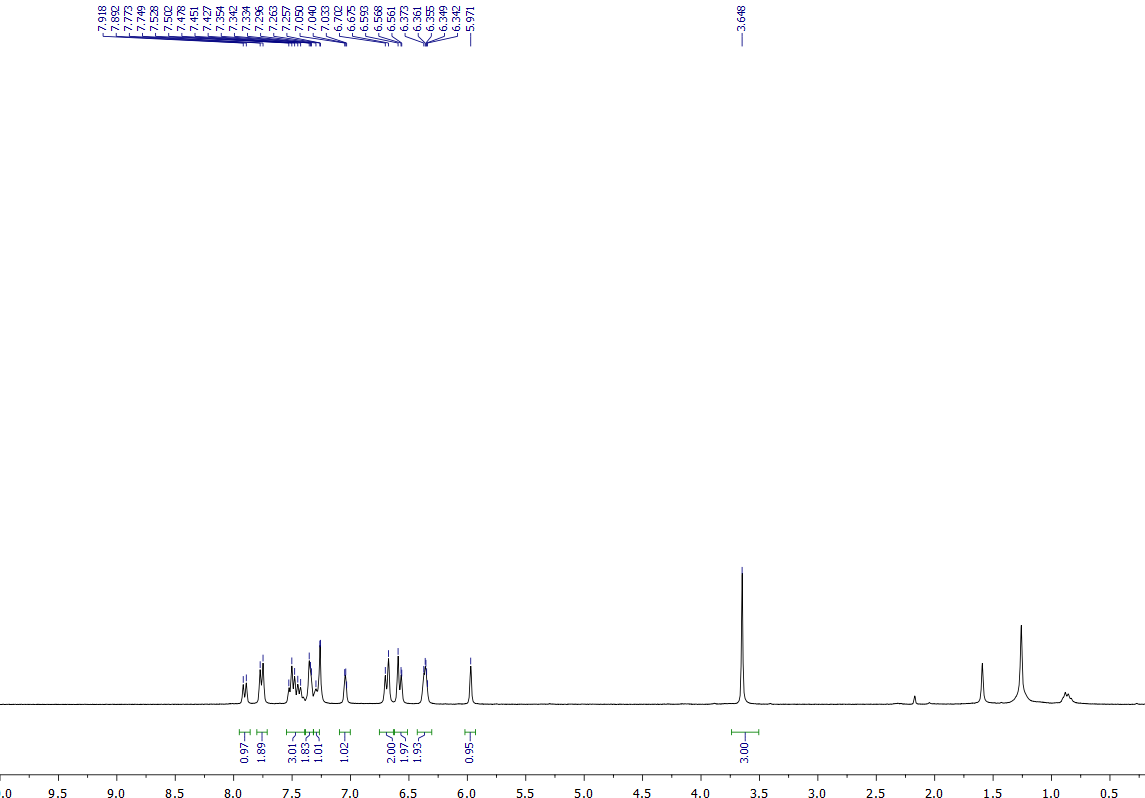

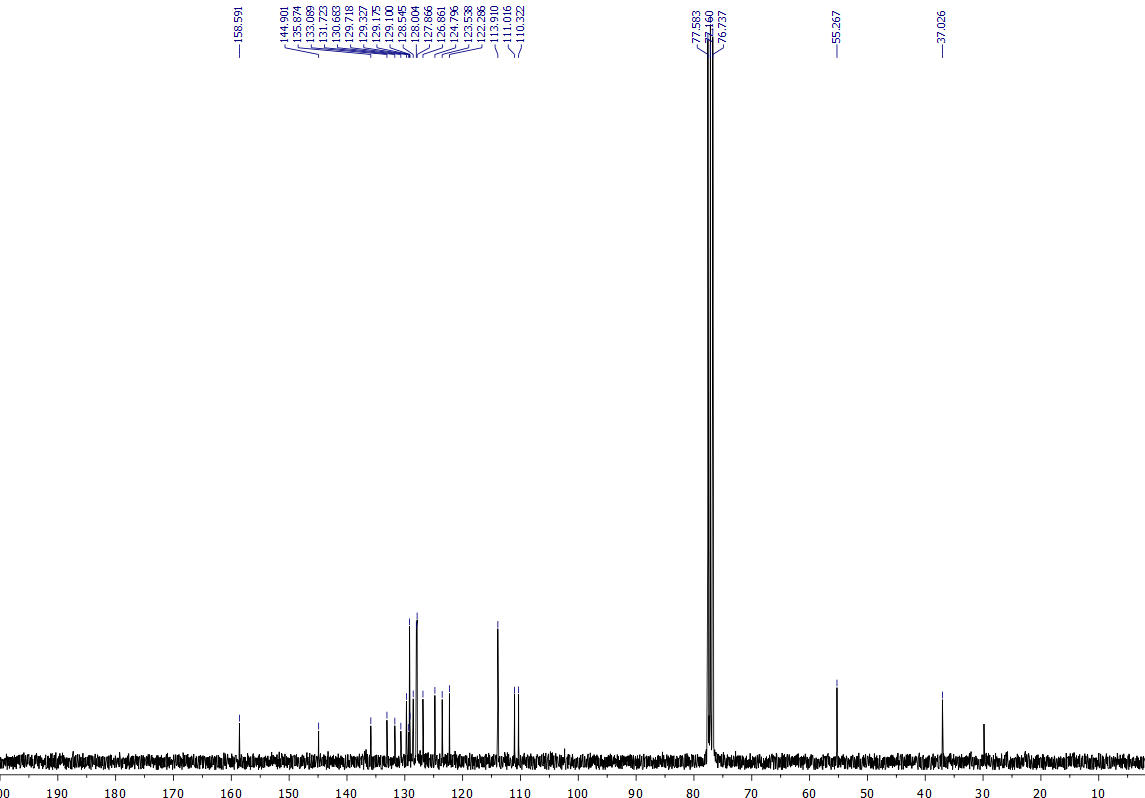


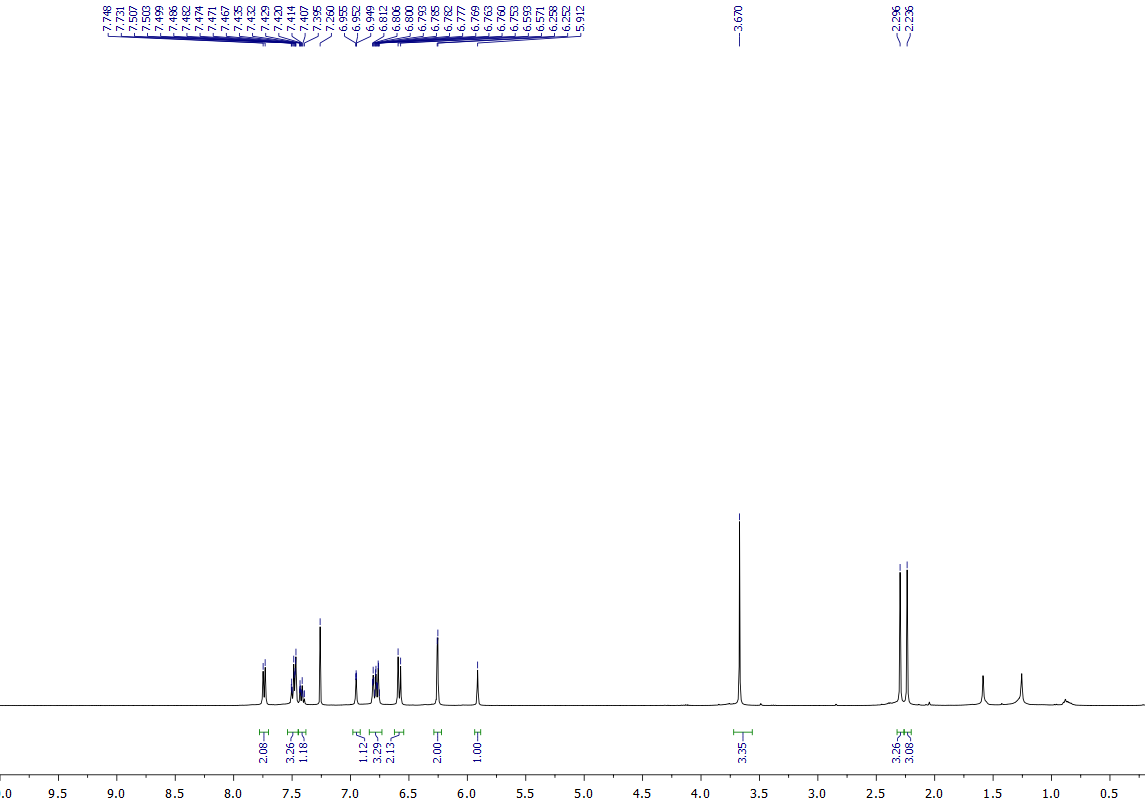

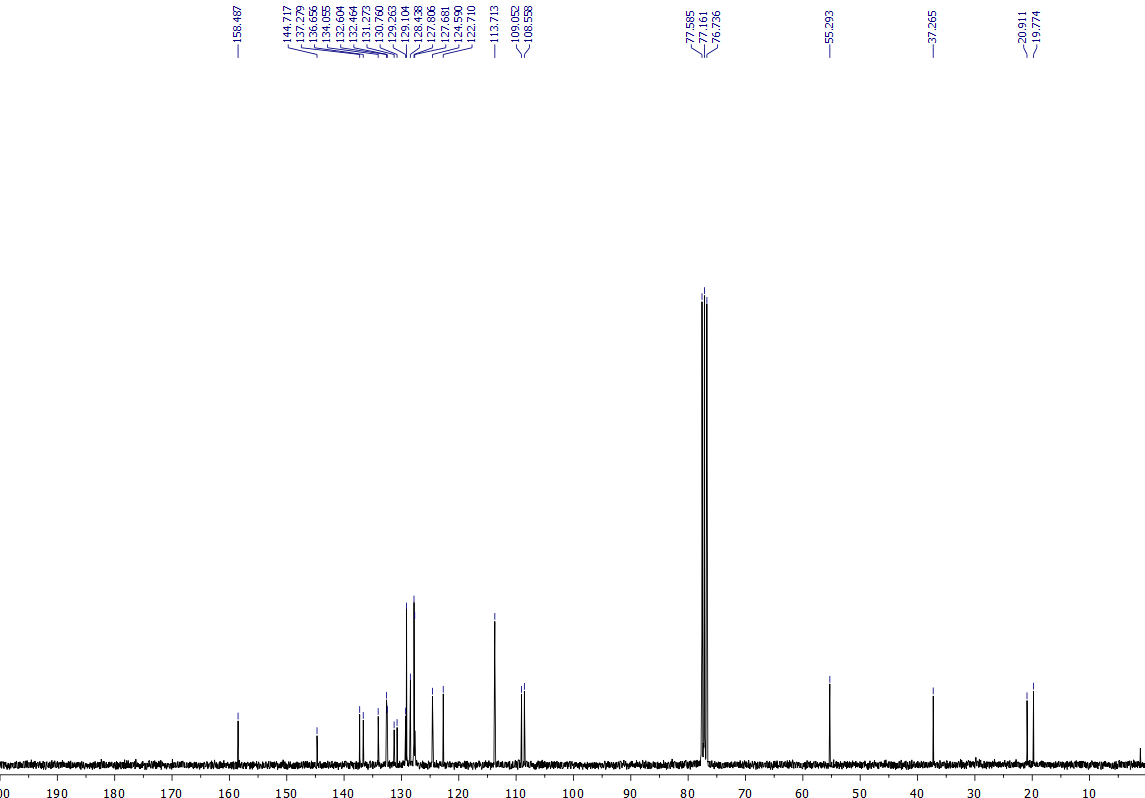


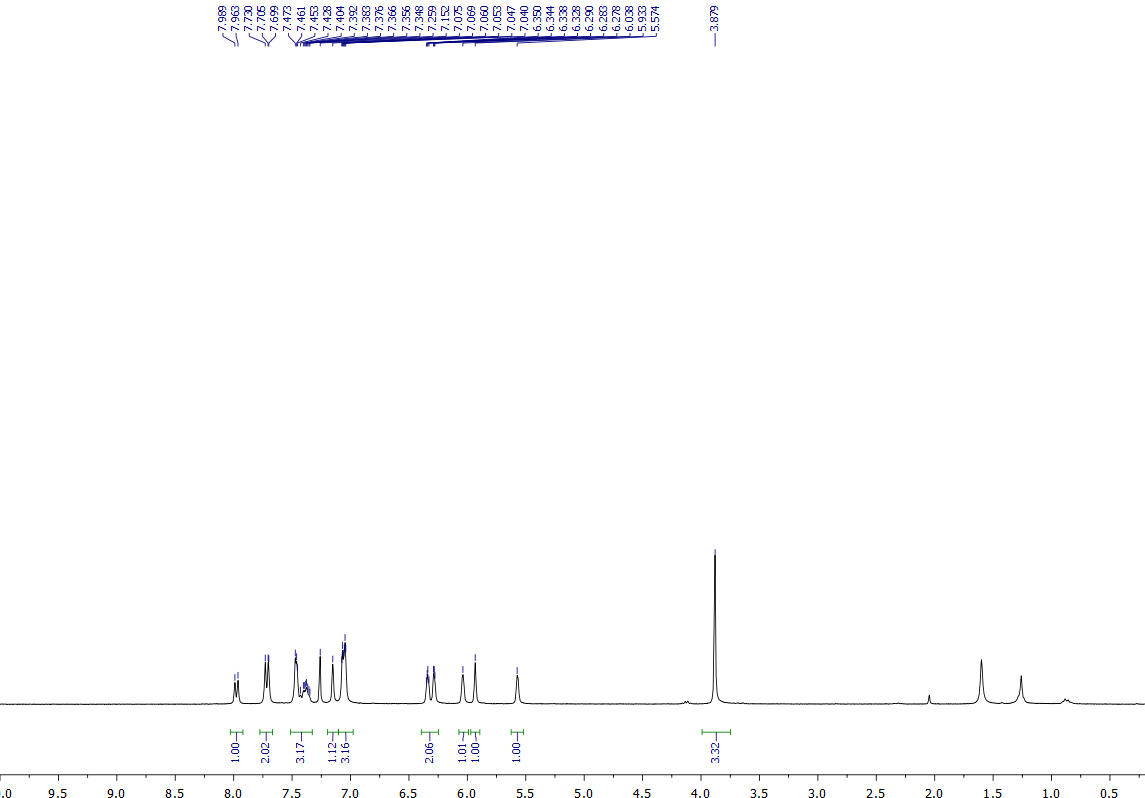

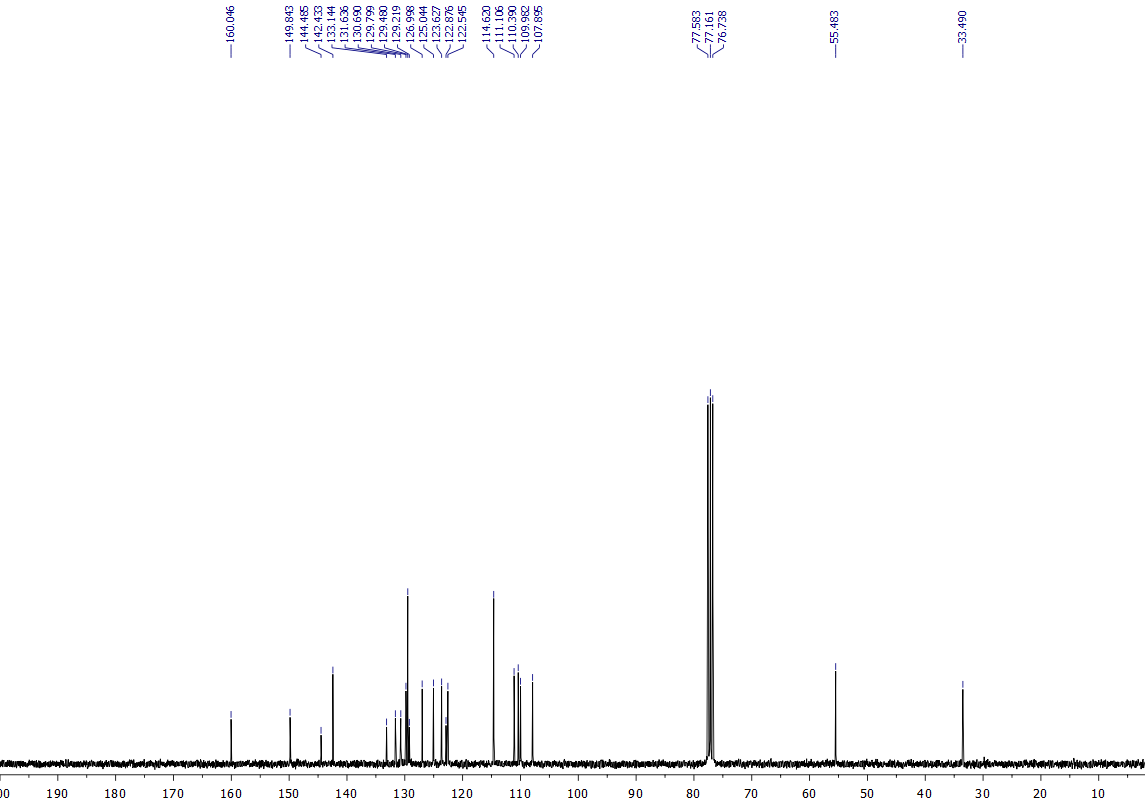


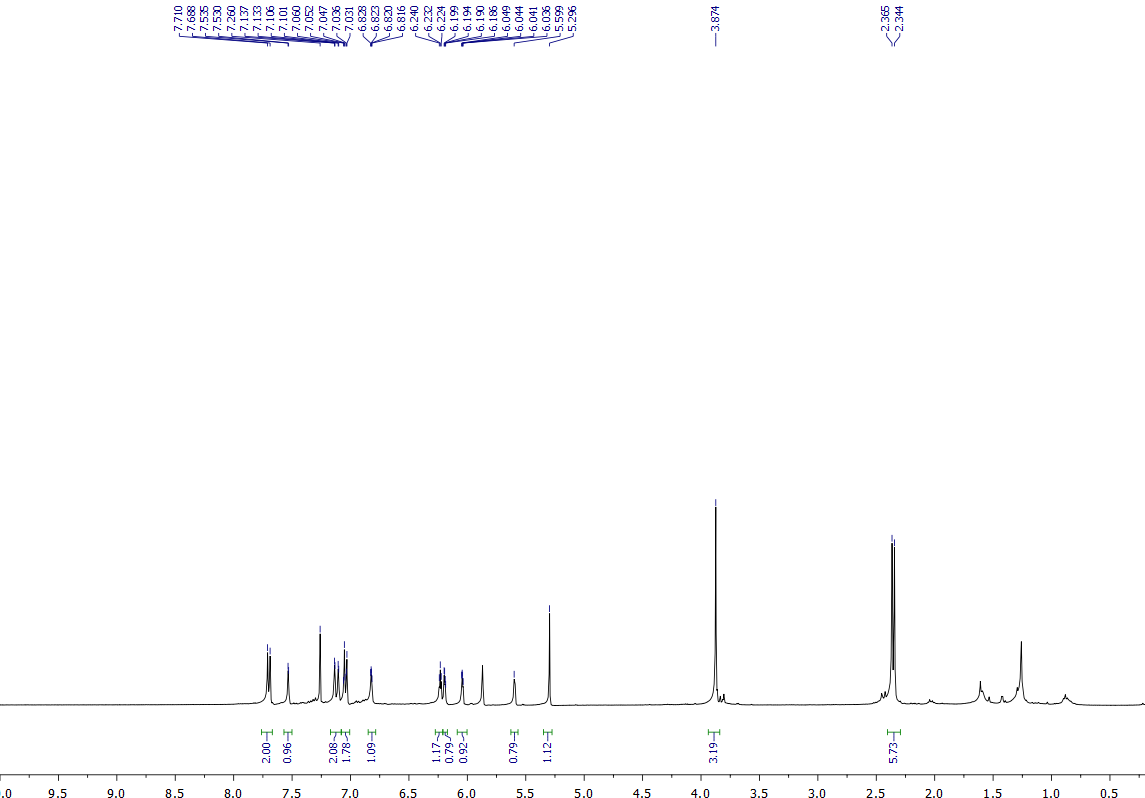


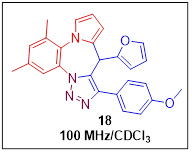

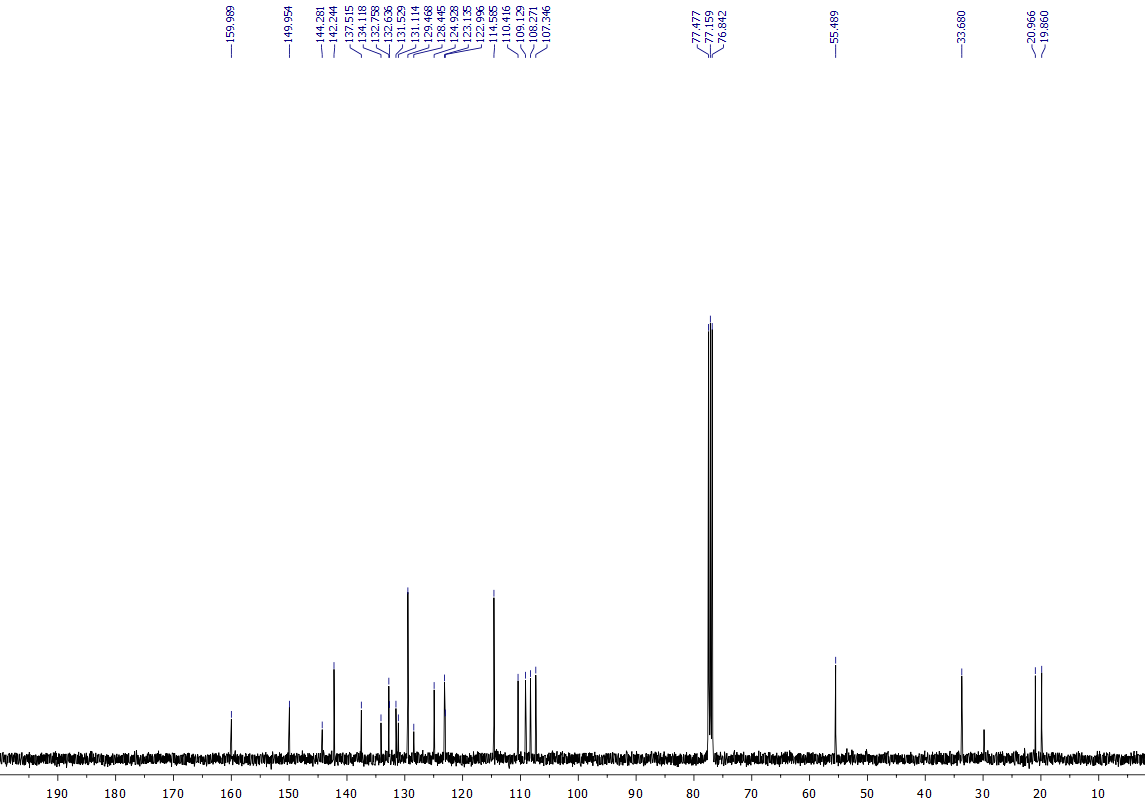

Supplement: Supplementary file 1 [file DataSheet1.docx]
